# Clinical parameters and emerging biomarkers of partial remission in pediatric type 1 diabetes: a systematic review

**DOI:** 10.3389/fendo.2026.1758848

**Published:** 2026-02-09

**Authors:** Camille Dikranian, Oumayma Hadara, Philippe A. Lysy

**Affiliations:** 1 Pôle EDIN, Institut de Recherche Expérimentale et Clinique, UCLouvain, Brussels, Belgium; 2 Specialized Pediatrics Service, Cliniques Universitaires Saint-Luc, Brussels, Belgium

**Keywords:** adolescents, autoimmunity, biomarkers, children, clinical factors, honeymoon phase, immune cells, partial remission

## Abstract

**Background:**

In type 1 diabetes (T1D), partial remission (PR) is a pivotal phase with preserved β-cell function, better glycemic stability, and reduced disease burden, and is as such a potential target for disease-modifying interventions. Identifying robust biomarkers of PR is critical for designing targeted therapies. This systematic review synthesizes current evidence from observational studies of biomarkers associated with PR in pediatric T1D.

**Methods:**

We searched the literature in PubMed, Scopus, and Embase (2009–2025), using strategies based on PICOS criteria. Investigated biomarkers covered multiple domains: anthropometric and clinical factors, continuous glucose monitoring metrics, HLA genotyping, immune cell and cytokine profiles, hormones, proteomics, and microRNAs. Eligible studies included observational cohorts of children and adolescents with newly diagnosed T1D. PR was defined as IDAA1c ≤9, HbA1c <7% with insulin requirement <0.5 IU/kg BW/day, or stimulated C-peptide ≥ 300pmol/L. Studies were selected according to PRISMA guidelines, and risk of bias was appraised using the Joanna Briggs Institute checklist.

**Results:**

Of 353 records, 39 studies including 9,368 patients met the inclusion criteria. Study populations ranged from 16 to 3,657 participants, with mean age of disease onset ranging from 7.0 to 13.8 years. Most studies (n=32) defined PR using IDAA1c. Routine clinical parameters and CGM-derived indices consistently distinguished remitters from non-remitters. Biological markers like immune signatures or proteomic profiles provided mechanistic insights into PR pathways. The methodological quality was moderate to high, though control of confounders and follow-up were incomplete.

**Conclusion:**

Standard-of-care biomarkers appear sufficient to identify PR and monitor its impact on glycemic outcomes. Emerging biological markers offer promising insights into the underlying mechanisms of PR. Well-powered studies are needed to clarify PR determinants and their therapeutic potential.

## 1 Introduction

Type 1 diabetes (T1D) is a chronic condition in which the body’s immune system destroys insulin-producing beta-cells (β-cells) located in the pancreas. This autoimmune disease can occur at any age, but it is most common in childhood and adolescence. It affects more than 1.5 million children worldwide (1), with an increasing incidence (2). The disease develops due to an interaction between genetic predisposition - strong association with HLA genotype, specifically DQB1 and DRB1 haplotypes - and environmental factors like viral infections or variations in the gut microbiome (3).

T1D progresses in three stages. In Stage 1, seroconversion to autoantibody positivity occurs and an autoimmune attack begins. This attack mainly involves T cells, which start to destroy pancreatic β-cells. In Stage 2, progressive loss of β-cell mass causes dysglycemia, or abnormal glucose metabolism, despite normal fasting glucose levels. In Stage 3, about 80% (4) of β-cells are destroyed, causing elevated fasting blood glucose and diabetes symptoms. Exogenous insulin is then necessary (2). The decline in β-cell mass is not linear, and the preclinical phase varies in duration among patients. Van Belle et al. proposed the “relapsing-remitting disease” model for T1D, arguing that the disease progression depends on a cyclical disruption and restoration of effector and Tregs balance (5).

**Figure 1:**
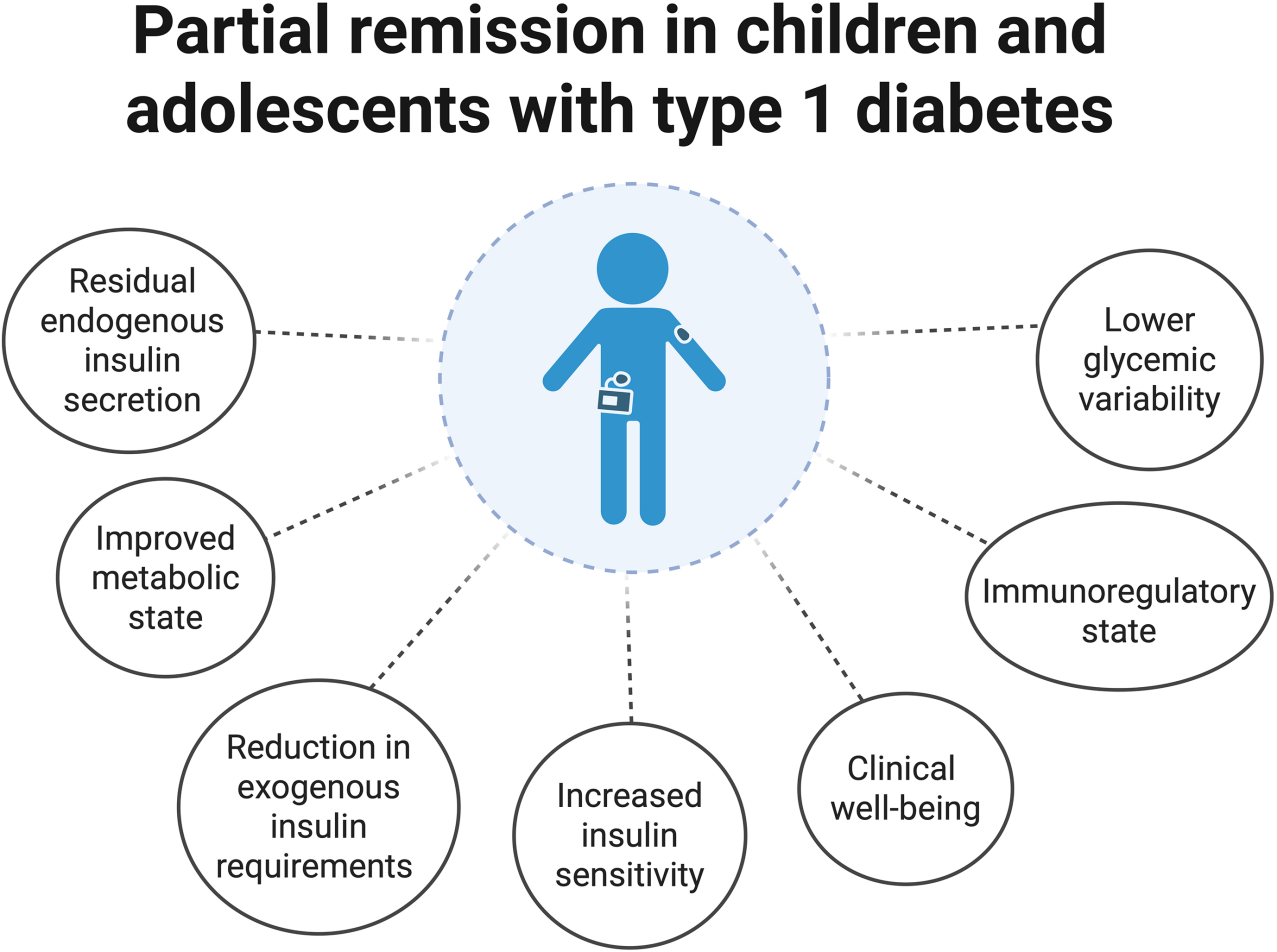
Graphical representation of clinical characteristics associated with partial remission in children and adolescents with type 1 diabetes. Created with BioRender.com.

Two-thirds of T1D patients experience the honeymoon phase or partial remission (PR) shortly after starting insulin therapy. This period is marked by a drop in insulin requirement and improved metabolic control (**Figure 1**). The duration varies from 3 to 24 months. Subsequently, patients become dependent on exogenous insulin again. Several hypotheses exist about the mechanisms underlying this disease period. Initiating insulin therapy can correct hyperglycemia by reducing metabolic stress and antigenicity, and promoting the recovery of remaining β-cells (6–8). During PR, the immune balance shifts from effector to regulatory cells, reducing the autoimmune attack on β-cells (9). Research has examined the interaction between glycemic control and immune response. During PR, modulating T-lymphocyte glucose metabolism could reduce the activation of pro-inflammatory immune subpopulations (6, 10).

In recent decades, several immunotherapies aimed at prolonging PR and slowing the decline in β-cell function have been evaluated. Teplizumab was approved by the Food and Drugs Administration (FDA) to slow T1D progression from Stage 2 to Stage 3 in patients aged 8 years and older (11). Teplizumab is a monoclonal antibody targeting CD3, a subset of the T-cell receptor complex essential for the activation of CD4^+^ and CD8^+^ T cells, which play a crucial role in the destruction of pancreatic β cells (12). While teplizumab has no yet been approved for stage 3 T1D, the PROTECT trial showed that twice-weekly intravenous treatment with teplizumab after diabetes onset was effective in preserving β-cell function, as assessed by AUC C-peptide levels during a mixed-meal tolerance test (MMTT) between baseline and 78 weeks post-diagnosis (13).

Other immunomodulatory therapies, such as abatacept (CTLA4-Ig), golimumab (anti-TNFα), rituximab (anti-CD20), alefacept (anti-CD2) and low-dose anti-thymocyte globulin, have shown variable results in preserving β-cell function (14, 15). However, robust conclusions about long-term efficacy and safety of these immunological interventions are difficult to draw since observed differences are often not sustained beyond the treatment period and the majority of clinical trials include small numbers of participants (12). Combining immunotherapies, sequencing different strategies, and adding interventions to improve β-cell function could enhance treatment efficacy (14, 15).

While no immunotherapy is currently approved for maintaining PR in T1D, there is evidence supporting the use of immunotherapy during the honeymoon phase, when there is still significant β-cell function. These therapies work through modulation of the autoimmune response driving β-cell destruction. T cell-directed therapies like teplizumab and abatacept inhibit autoreactive T cell activation and promote regulatory T cell expansion. Anti-cytokine agents like golimumab inhibit inflammatory cytokines like Tumor Necrosis Factor alpha (TNFα), which are implicated in β-cell apoptosis and immune activation (12).

Moreover, *post hoc* analyses of trials including individuals with stage 3 T1D suggest that immune interventions such as teplizumab are more effective in specific patient subgroups. These are characterized by features associated with partial remission, including higher baseline C-peptide levels, lower baseline insulin requirements and HbA1c, and a shorter duration since diagnosis (16). We believe that identifying biomarkers and clinical factors associated with PR is essential for earlier, more personalized patient management by selecting the patients most likely to benefit from interventions and assessing treatment response (17).

This review summarizes current evidence from observational studies investigating biomarkers associated with PR in pediatric T1D.

## 2 Methods

### 2.1 Protocol

We conducted this review following the Preferred Reporting Items for Systematic Reviews and Meta-Analyses (PRISMA) guidelines (18).

### 2.2 Eligibility criteria

We selected studies using the PICOS framework. The inclusion criteria were as follows:

- Population: Children and adolescents with newly diagnosed T1D.
- Intervention/Exposure: Assessment of anthropometric/clinical factors and biomarkers including immune cell subsets, glycemic parameters, proteomic profiles, HLA genotypes, cytokines, hormones, and microRNAs.
- Outcomes: PR occurrence defined by one of the following criteria (19, 20):
  ○ Daily insulin requirement <0.5 units/kg of body weight (BW)/day with HbA1c <7%;
  ○Stimulated C-peptide ≥300pmol/L;
  ○Stimulated C-peptide >300pmol/L;
  ○IDAA1c ≤9;
  ○IDAA1c <9.
- Study design: Prospective or retrospective observational studies published in English between 2009 and 2025, with full-text available.

Studies using alternative definitions of remission, unavailable in English, or including adult or T2D populations without stratifying the results were excluded.

### 2.3 Search

We conducted a literature search using tailored strategies for each database. Using the PICOS framework, we created the following core search string by combining relevant keywords with Boolean operators: (“pediatric diabetes mellitus, type 1” OR “pediatric type 1 diabetes” OR “pediatric new-onset type 1 diabetes”) AND (“partial remission” OR “honeymoon phase” OR “remission phase”) AND (biomarkers OR “immune cells” OR “glycemic parameters” OR proteomics OR autoantibodies OR cytokines OR hormones OR microRNA). We first reviewed the existing literature on PR in pediatric T1D to inform the construction of these search equations and identify relevant categories of biomarkers in this field. The equations were adapted to the specific indexing systems of PubMed, Scopus, and Embase. Both title/abstract and controlled vocabulary terms (MeSH, Emtree) were used. The search, performed in October 2025, was restricted to studies published since 2009. The full search strategies for each database are provided in **Supplementary Data 1**.

### 2.4 Study selection

All records from the database searches were imported into a reference management software, where duplicates were removed. Then, articles were screened according to the predefined PICOS criteria and language restrictions. Two researchers performed the search independently, resolving disagreements through discussion until consensus was reached.

Two reviewers conducted data extraction from the included records independently using a standardized form. The extracted information included the following: study design; population characteristics; biomarker categories and detection methods; definitions of PR; follow-up duration. When the mean age was unavailable, the following formula was used to estimate it based on median age and percentiles: mean ≈ $\frac{P25+2(median)+P75}{4}$. Mean age was unavailable in one publication (21). Any discrepancies between the reviewers were resolved through deliberation until an agreement was reached.

A summary table was constructed for each study to synthesize cohort characteristics and PR definitions and document the methods used for biomarker identification. These data are presented in **Supplementary Table 1**.

### 2.5 Risk of bias in individual studies

#### 2.5.1 The Joanna Briggs Institute critical appraisal checklist

We used the JBI Critical Appraisal Checklist for Cohort Studies to evaluate the methodological quality of the observational studies included in our review (22). The checklist has eleven items designed to evaluate potential sources of bias and methodological rigor of the study. The first items address the comparability of the study groups and whether they were recruited from the same population. They also address whether exposures are measured in a valid and reliable way. Other items examine whether confounders were identified and managed appropriately, and whether outcomes were absent at baseline. Other criteria focus on the validity and reliability of outcome measurements, sufficiency and completeness of follow-ups, and strategies for managing incomplete data. The final item evaluates the appropriateness of the statistical analyses. In line with JBI guidance, each item is considered individually without producing an aggregated score. Our findings are presented transparently in **Supplementary Table 2**, which displays item-by-item judgments for each study.

## 3 Results

### 3.1 Study selection

**Figure 2:**
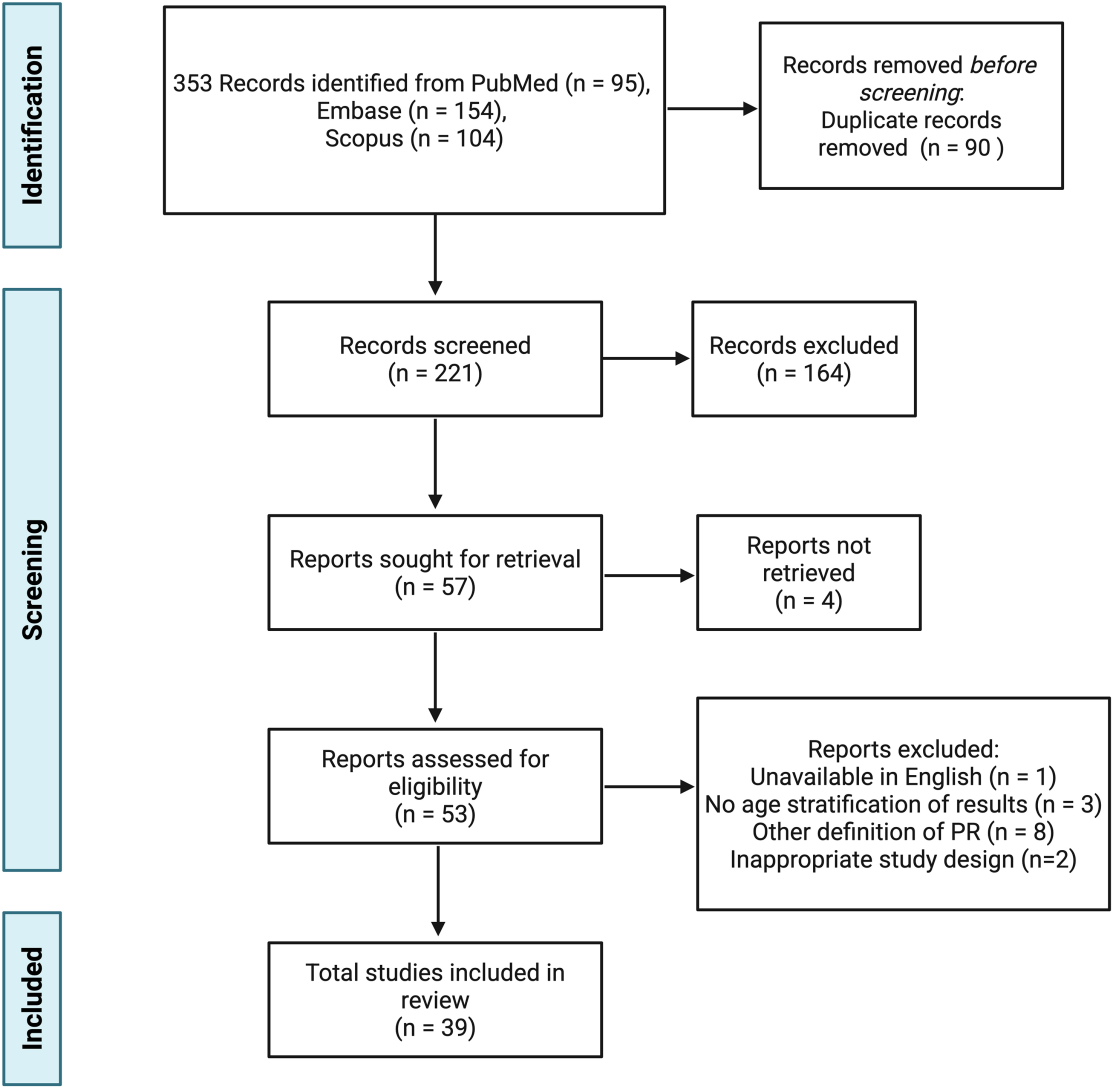
PRISMA flow diagram illustrating the article selection process. Created with BioRender.com.

The literature search yielded 353 records from PubMed (n = 95), Scopus (n = 104), and Embase (n = 154). After removing 90 duplicates using Zotero, 221 articles remained for title and abstract screening. Of these, 164 did not meet the predefined PICOS criteria. Four records were unavailable in portable document format (PDF). Of the records assessed for eligibility, one was unavailable in English, three included adult participants without age stratification, eight used a different PR definition, and two were excluded due to their study design. Ultimately, 39 articles were included. **Figure 2** details the selection process.

### 3.2 Risk of bias within studies

The 39 evaluated studies had moderate risk of bias. Most studies used well-standardized exposure and outcome measures and appropriate statistical analyses, but methodological limitations explain the intermediate rating. Most studies did not use formal multiple imputation or perform detailed sensitivity analyses to evaluate the effects of attrition regarding data completeness (items 9 and 10). Authors often used complete-case analyses or repeated measure models. These assume missing data are random. Alternatively, they excluded “non-adherent” subjects, without exploring reasons for loss or performing multiple imputations.

The most frequently identified confounders for items 4 and 5 were as follows: age at diagnosis, gender, BMI/BMI-SDS, HbA1c at diagnosis, diabetic ketoacidosis (DKA), insulin dose, measures of β-cell secretion (C-peptide), and autoantibodies. Repeated analytical strategies include the following:

- Multivariate regression adjustments (logistic/linear);
- Using linear mixed models and generalized linear mixed models (GLMM) for longitudinal series;
- Stratifications/sub-analyses (*e.g*., by age or center);
- Matching in some studies.

We assigned ‘YES’ to item 5 in the JBI grid when a study presented multivariate analyses including most of the major clinical cofactors listed above.

However, some important confounders were often overlooked or inadequately considered, such as pubertal status, direct measurements of insulin sensitivity/resistance (*e.g.*, clamp, HOMA), food intake and socio-economic status. Omitting these factors can introduce residual bias, especially in studies examining the ‘honeymoon’ period’s occurrence and duration in children.

### 3.3 Study characteristics

This systematic review included a total of 9,368 patients. The sample sizes across the analyzed clinical studies ranged from 16 to 3,657 participants, with mean ages between 7.0 and 13.8 years. Age at disease onset was < 18 years in 37 studies, < 19 years in one study and ≤ 25 years in one study; in the latter, the median age at onset was 10.3 years, supporting its inclusion despite the higher upper age limit (23). All patients were recruited in hospital or diabetes clinic settings. All studies were observational cohorts, 10 of which were retrospective. The IDAA1c score was used to define PR in 32/39 studies. Two studies used both IDAA1c and stimulated C-peptide ≥300pmol/L (24, 25). One study used stimulated C-peptide ≥300pmol/L alone (26). Six studies used both HbA1c levels <7% and insulin requirements <0.5 IU/kg BW/day (27–32). One study used both IDAA1c and the combination of HbA1c levels <7% and insulin requirements <0.5 IU/kg BW/day (33). The duration of follow-up ranged from three to 72 months. A follow-up period of at least 12 months was reported in 36 records.

**Figure 3:**
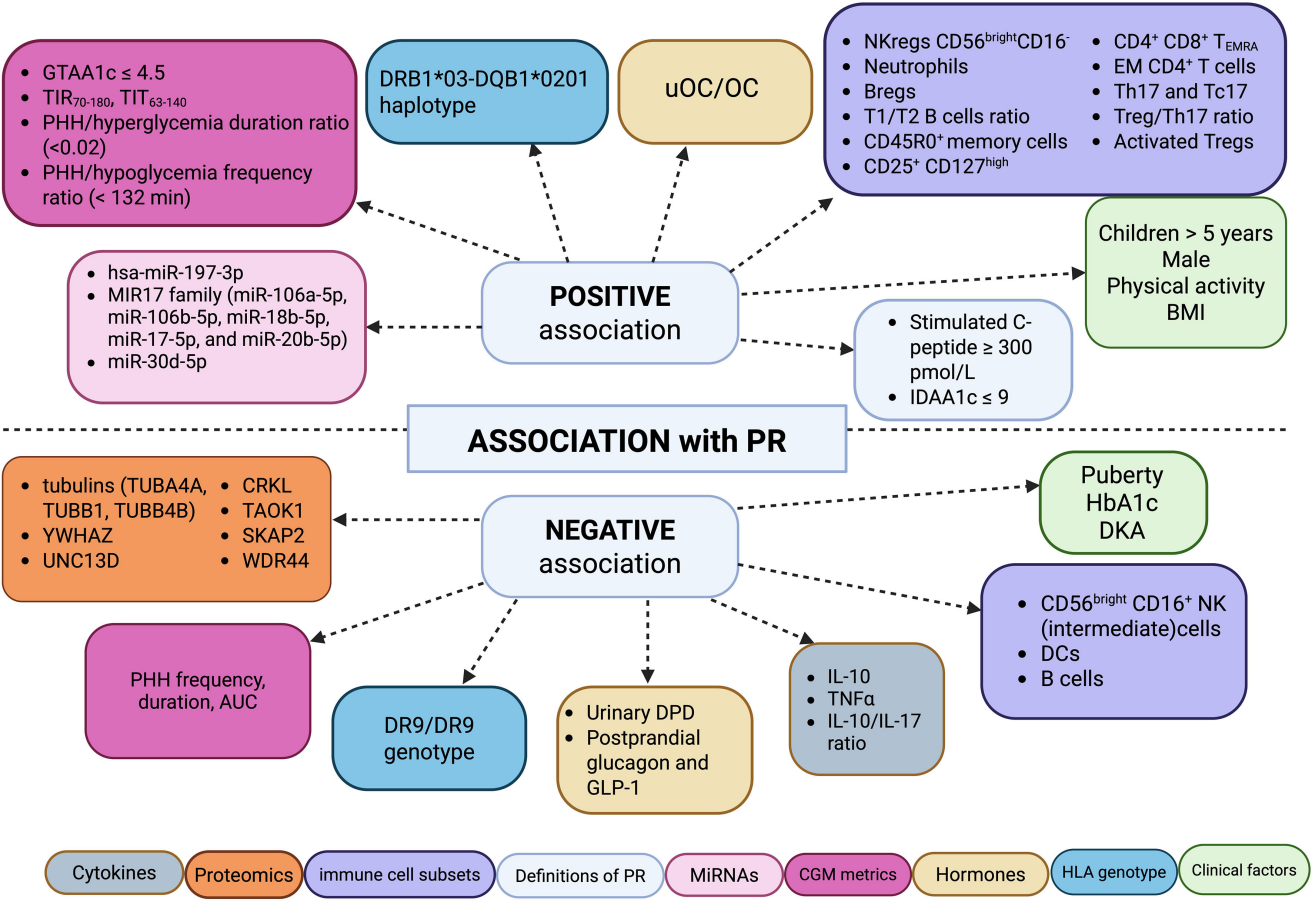
Graphical representation of markers positively and negatively associated with partial remission in pediatric patients with type 1 diabetes. Created with BioRender.com.

The included studies were categorized by the type of biomarker investigated: PR definitions (20, 34), anthropometric data (23, 25, 28, 29, 35–44), CGM metrics (45–48), HLA genotyping (23, 26), variations in immune cells (21, 32, 33, 49–53), hormones (24, 30, 31, 54–56), proteomics (57), and microRNAs (58–60) (**Figure 3**).

## 4 Necessity and relevance of defining partial remission

Clinically, PR is associated with preserved β-cell function, reduced disease burden, and a lower risk of long-term complications. Patients in honeymoon phase demonstrate improved early glycemic control and reduced glycemic variability (e.g., standard deviation, coefficient of variability, and percentage of normoglycemia) (46, 61).

The Diabetes Control and Complications Trial (DCCT) demonstrated that maintaining an HbA1c level of 7% with intensive therapy can significantly lower the risk of early microvascular complications like retinopathy, nephropathy, and neuropathy, by 35 to 76% (62, 63). The Epidemiology of Diabetes Interventions and Complications (EDIC) Research Group demonstrated a 58% reduction in major cardiovascular events after a 18-year follow-up through intensive glycemic control (63). The introduction and widespread use of CGM has brought forward the concept of glycemic variability, which may complement mean HbA1c in assessing the risk of diabetes-related complications. Longitudinal studies are still lacking, but reduced glycemic variability during PR (47, 61) could decrease microvascular and macrovascular diabetes complications (64). Recent longitudinal studies demonstrated that patients with T1D who experience PR exhibit distinct C-peptide secretion dynamics, reflecting endogenous insulin secretion. According to Shi et al., three years after diabetes onset, 71% of patients who experienced PR retain residual β-cell function, compared to only 11% of those who did not (65). A longitudinal Finnish study showed that persistent C-peptide secretion is common years after diagnosis, especially among those who experience PR. This is linked to improved metabolic outcomes and a reduced risk of microvascular complications (66). The likelihood of improved diabetes outcomes underlines the importance of attempting to prolong the partial remission period.

The development of disease-modifying therapies (13, 67–72) has made it clinically important to identify the PR phase since the efficacy of immunotherapies strongly depends on the timing of intervention (12, 68, 73). Some experts hypothesize that remitters may respond better to immunotherapy because the mechanisms underlying PR could provide a permissive environment for immunologic intervention (74). Early identification of remitters would enable optimized therapeutic stratification and facilitate the inclusion of patients into clinical trials targeting this specific window.

T1D is a heterogeneous disease, as Dayan et al. emphasized (75). Patients differ in their residual insulin production and the immunopathological mechanisms driving β-cell decline. The Exeter group’s analyses of pancreatic tissue from patients recently diagnosed with T1D have demonstrated age-related differences in the severity of insulitis; compared to patients diagnosed before age 7, those diagnosed after age 13 displayed a milder infiltrate with fewer CD20^+^ cells (76). These histopathological findings have not been correlated with clinically identified “slow progressors” yet (77, 78). This subgroup may only require moderate immunomodulation. The observed heterogeneity in disease mechanisms underscores the necessity of multimodal immunotherapeutic approaches tailored to the appropriate patient at the appropriate time rather than a uniform “one size fits all” strategy (75). However, proof-of-concept studies have not yet validated such multimodal approaches. Adaptive platform trials, such as T1D-Plus, reflect this rationale. These trials aim to test multiple interventions within broad patient groups. This approach moves toward more widely applicable therapeutic strategies (Dayan et al., T1D-Plus trial documentation).

## 5 What about complete remission?

Complete remission (CR) is defined as zero daily insulin requirements (0 IU/kg BW/day) and optimal glycemic control (HbA1c < 7%). Similarly to PR, it is a transient phenomenon. The prevalence of CR among newly diagnosed T1D patients is estimated to be below 3%, making it substantially less frequent than PR (79, 80). Although robust evidence is lacking due to the low frequency of this condition, factors associated with the occurrence of CR appear to be similar to those linked to PR. These may include higher serum C-peptide levels, absence of DKA, and lower HbA1c levels at disease onset. Available data also suggest that earlier diagnosis of T1D increases the likelihood of CR, thereby supporting the rationale for T1D screening to capture early stages of the disease (79). Given the limited body of literature on this topic, this review focuses on the concept of PR.

## 6 Assessment of residual β-cell function and clinical definitions of partial remission

### 6.1 Historical definitions

Since Jackson et al. first introduced the concept of PR in T1D (81), its definition has varied. Early studies defined PR as having low insulin requirements, typically ≤0.5 IU/kg BW/day, and sometimes ≤0.3 IU/kg BW/day. This definition was used alone or combined with a HbA1c level ≤ 7.5%. This definition is intuitive but confuses the disease state with the treatment strategy. The insulin dosage is a decision variable shaped by patient-level factors (*e.g*., age, pubertal status, BMI, life context, and support) and center-level factors (*e.g*., therapeutic targets, protocol, education, and technology uptake). It is too contingent for a solid PR definition. Dose-based definitions misclassify patients as “in remission” when they receive low doses, regardless of other factors (20). HbA1c alone is problematic because its level is influenced by insulin doses, and stabilization takes four to six weeks after diagnosis. This has prompted the search for less treatment-dependent indicators, including C-peptide measurements, HbA1c levels combined with insulin doses at varying thresholds, and composite indices that adjust insulin doses according to HbA1c levels. In 2018, the International Society for Pediatric and Adolescents Diabetes (ISPAD) defined PR as having an insulin dose <0.5 IU/kg BW/day and an HbA1c level <7% (19).

### 6.2 C-peptide

C-peptide is the gold standard for measuring residual β-cell function (82). It has a longer half-life than insulin and is secreted at equimolar concentrations. Unlike insulin, C-peptide is not subject to hepatic clearance, and quantification by immunoassays shows no cross-reactivity with exogenous insulin (83). The measurement of C-peptide area under the curve (AUC) or peak stimulated C-peptide during mixed-meal tolerance tests (MMTT) is the preferred method for evaluating β-cell function in interventional studies (83). The most common threshold for PR in clinical studies is a stimulated C-peptide level ≥300pmol/L. This level is widely recognized as corresponding to clinically meaningful residual β-cell function (20, 34). However, C-peptide has significant limitations as a biomarker of PR (84, 85). C-peptide variability as biomarker stems from heterogeneous cutoff values, assay platforms (*e.g.*, chemiluminescence vs. fluoro-immunoassays), and cohort-related factors. Reported serum C-peptide positivity varies widely across studies due to these factors (83, 86). MMTT also depends on an intact incretin axis (GIP/GLP-1) (87), which is impaired in individuals with newly diagnosed T1D (55). This can underestimate residual β-cell function (85, 88). Furthermore, stimulated C-peptide responses do not consistently correlate with clinical outcomes (47, 89, 90) because glycemic control during PR depends on both insulin secretion and sensitivity. Insulin sensitivity varies with age, BMI, sex, and pubertal status (85). Only patients with high peak C-peptide levels (>400pmol/L) exhibit significant β-cell glucose responsiveness (91, 92).

C-peptide assays cannot distinguish between β-cell mass and function (86). Proinsulin/C-peptide ratios show that residual insulin secretion and functionality are not necessarily linked due to defective post-translational processing (55, 93). These findings reveal a discrepancy between endogenous insulin secretion and glycemic homeostasis. This can be partly explained by individual variations in insulin sensitivity and β-cell glucose responsiveness during the first year post-diagnosis (86, 94, 95).

### 6.3 IDAA1c

PR may also be defined using the insulin dose-adjusted HbA1c (IDAA1c) score ≤9. This definition, proposed by Mortensen et al. (2009), combines HbA1c with daily insulin requirements according to the formula: IDAA1c = HbA1c (%) + [4 × insulin dose (IU/kg/day)] (20). In the Hvidoere cohort of 275 children and adolescents with newly diagnosed T1D, high correlation was found between β-cell function (assessed by stimulated C-peptide) and IDAA1c level ≤ 9. Six months post-diagnosis, IDAA1c moderately predicted stimulated C-peptide values at 6 and 12 months (R^2^ = 0.30 and 0.31, respectively) (20). The score reflects the impact of changes in β-cell function on glycemic homeostasis. It is less costly, less invasive, and less burdensome than direct C-peptide measurement. It has been validated in several cohort studies (20, 34, 37) and is recognized as an international standard (96).

Clinical models are the most common definition of PR, but they have limitations. The correlation between IDAA1c and stimulated C-peptide becomes less sensitive and specific with age (20, 34, 37) and tends to underestimate C-peptide levels in children with IDAA1c >9 (84, 90). This is especially true for patients over 10 years old because IDAA1c does not provide direct information about the relative contribution of residual insulin secretion and increased insulin resistance (20, 34). As mentioned above, the score depends on HbA1c and insulin dose, both of which are influenced by confounding factors (treatment guidelines, physician practices, etc.) (46). Another limitation of insulin-dose-based criteria is that insulin dose can be reduced by insulin pump therapy, some adjunctive medications and low-carbohydrate diets. In addition, reported insulin doses are approximations as they are self-reported and do not account for correction boluses. However, in patients treated with continuous subcutaneous insulin infusion, insulin dose requirements are reliable when devices are connected to sharing platforms.

## 7 Biomarkers of partial remission: within the standard of care

### 7.1 CGM metrics

#### 7.1.1 Glycemic target-adjusted HbA1c

Since its introduction into routine care, CGM has demonstrated glycemic variability as a marker of diabetes. In 2018, our group proposed the use of simple glycemic variability parameters for defining PR (46). Multivariate analysis revealed that HbA1c levels and time in normoglycemia were key to predicting PR using the IDAA1c criterion. The GTAA1c was proposed: GTAA1c = HbA1c (%) − (3 × [% normoglycemia (70–180 mg/dL)]). A GTAA1c level of ≤ 4.5 predicted PR with 72.3% sensitivity and 92% specificity. The score demonstrated high specificity in patients <5 years old and high sensitivity in those >10 years.

#### 7.1.2 Continuous glucose monitoring

Our group argued that CGM data provide robust and sufficient PR markers in pediatric T1D (47). As Buckingham et al. reported (84), residual endogenous insulin secretion estimates only partially reflect glycemic homeostasis. In the DIATAG (DIAbetes TAGging) consortium, measures of residual C-peptide secretion correlated well with each other (p <0.001) but only weakly with clinical parameters (IDAA1c, HbA1c, and total daily insulin dose). By contrast, strong correlations were found between clinical parameters and CGM-derived metrics. Time in range (TIR_70-180_), time in target range (TIT_63-140_), and time above range (TAR_>180_) had the strongest correlations with HbA1c levels (p <0.0001) and IDAA1c (p <0.0001). The correlation between CGM-derived metrics and estimates of endogenous insulin secretion was moderate. The highest correlation with C-peptide estimate (CPEP_est_) was seen in TAR_>180_ and TIR_70-180_ (r^2^ = 0.13 and r^2^ = 0.22, respectively; p <0.01), whereas time below range (TBR_<70_) did not correlate (47).

CGM analysis enabled a detailed characterization of glycemic homeostasis. Remitters spent more time in TIR_70–180_ and TIT_63–140_ and less time in hyperglycemia throughout the day. Compared with non-remitters, they displayed lower glycemic variability within and between days. Early morning was the most sensitive time to distinguish remitters from non-remitters, particularly through TIT_63–140_ and TAR_>180_. Low-grade hypoglycemia in the early morning (0400h-0700h) identified remitters, with TBR_<70, 63–70_ being a clinically meaningful marker of residual β-cell function. In contrast, Grade II hypoglycemia (GIIH_<54_) correlated negatively with residual β-cell secretion, confirming that dependence on exogenous insulin promotes severe hypoglycemia in recently diagnosed patients (47). Among 65 pediatric T1D patients, Addala et al. found in remitters a significant increase in time spent between 54 to 70mg/dL over a 24-hour period (p = 0.04). This increase was not found at nighttime (0000h-0600h). The difference was driven mostly by the time spent between 65 and 70mg/dL (p = 0.02). No correlation was found between remission status and clinically severe hypoglycemia (<54mg/dL) (45). Detailed analysis of CGM data combined with clinical parameters allowed our team to define different remission subgroups. This approach refined the characterization of the overlap observed in intermediate HbA1c values between remitters and non-remitters, by identifying four distinct “glucotypes” that reflect the progressive loss of glycemic homeostasis during the first year after diagnosis. These glucotypes differed significantly from one another in terms of clinical parameters (IDAA1c, HbA1c, TDD) and CGM measures (47), as confirmed by Zhong et al. in adult patients (97).

Recent machine learning (ML) approaches provide complementary evidence that strengthens the usefulness of CGM data for identifying the honeymoon phase. Satheeskumar et al. demonstrated that advanced ML models can detect the honeymoon phase with high accuracy (up to 91%), using features from CGM, glucose management indicator (GMI) reports, insulin dosing patterns, and HbA1c trends (98). These models could outperform traditional clinical assessments by capturing subtle temporal patterns and glucose fluctuations indicative of PR.

#### 7.1.3 Post-hypoglycemia hyperglycemia

Clinical observations suggest that PR is associated with reduced glycemic variability (GV), which is influenced by insulin dosing, carbohydrate intake, and physical activity. We focused on these parameters to implement CGM metrics related to PR. Analyzing specific CGM patterns during the first year post-T1D diagnosis introduced the concept of post-hypoglycemia hyperglycemia (PHH), defined as hyperglycemia (>160mg/dL) within two hours after hypoglycemia (<60mg/dL) (48, 99). PHH formalizes the concept of rebound hyperglycemia (100).

PHH parameters were expressed as PHH frequency, PHH_AUC_, mean PHH duration, PHH/hyperglycemia duration ratio, and PHH/hypoglycemia frequency ratio. Of the 194 CGM recordings from 66 pediatric patients, the PHH parameters distinguished remitters from non-remitters (p <0.001). All of the studied PHH parameters were able to predict a patient’s remission status (all p-values <0.05). The PHH/hyperglycemia duration ratio (<0.02) was the most sensitive marker (Sn 86%, Sp 68%); the PHH/hypoglycemia frequency ratio (<132 min) was the most specific (Sn 76%, Sp 74%). Remitters experienced fewer PHH events, of shorter duration, and smaller AUC (all p <0.001) versus non-remitters. Non-remitters experienced more frequent PHH during the day and evening, with no differences observed at night or in the early morning. The largest PHH AUCs occurred overnight. However, remitters had significantly smaller values than non-remitters. PHH parameters were strongly correlated with CGM-derived measures, but only weakly/moderately with residual β-cell secretion. PHH measures confirmed the progressive increase in glycemic variability across the CGM-derived glucotypes. Patients with borderline IDAA1c values could be grouped into glucotypes 2 and 3, supporting the role of PHH parameters as reliable markers of glycemic homeostasis.

Hypoglycemia frequency, glycemic variability and PHH parameters distinguished three patient groups, each with specific variability and PHH parameter combinations:

- Group 1 (mainly remitters): CV <36%, stable T1D, low PHH/hypoglycemia frequency ratio (<0.25), and very low PHH AUC (<25 000);
- Group 2 (mainly non-remitters): CV >36%, variable PHH/hypoglycemia frequency ratio (0.1–0.5), and low-to-intermediate PHH AUC (<60,000);
- Group 3 (mainly non-remitters): very rare hypoglycemia, often followed by PHH, PHH/hypoglycemia frequency ratio >0.4, and PHH AUC >60 000.

Throughout the first year, more than half of the patients (53%) stayed in the same group, but some shifted. However, no transition was observed from groups 2 or 3 back to Group 1. Most Group 2 patients often overtreated their hypoglycemia when trying to reach a normoglycemic state, resulting in short and frequent PHH, thus high glycemic variability. Therapeutic education focusing on managing hypoglycemia and targeting a PHH/Hypoglycemia frequency ratio <0.25 may improve disease control.

CGM-derived markers for PR offer new insights into this critical period. Rather than viewing PR as a dichotomous event, it is now understood that deterioration of glycemic homeostasis during the first year after diagnosis follows a continuum. This continuum begins with daytime imbalance and continues at night, causing increased variability, hyperglycemia, and eventually Grade II hypoglycemia. CGM-derived glucotypes help stratify patients with intermediate HbA1c values (48).

#### 7.1.4 Anthropometric data/clinical factors

##### 7.1.4.1 Sex

Anthropometric and clinical factors influence the occurrence and duration of PR in T1D. Sex-related differences were reported: girls are less likely to enter PR. In the DPV cohort, boys had higher odds than girls of entering PR (adjusted OR 1.41) (37). Marino et al. found that male sex was associated with lower odds of non-remission (OR 0.51) and the non-remitter group included a higher proportion of females (59.3% vs. 43.0%) (38). Wong et al. reported a 2.2-fold higher relative risk of non-remission among female patients; this difference remained significant in multivariate regression (p = 0.003) (40). In a large Chinese cohort including 186 T1D children, male sex was independently associated with a higher likelihood of entering PR (aOR 2.42) (25). In a cohort of 99 children with new-onset T1D, Emet et al. reported that boys had an adjusted odds ratio of 3.8 for entering PR; in multivariate analysis, male sex was the sole predictor of PR (36). Yet, the IDAA1c score makes it hard to interpret this observation because it does not distinguish between insulin secretion and insulin sensitivity (37). The gender difference may reflect higher insulin resistance in girls, who typically enter puberty earlier and are thus exposed to pubertal hormones for a longer time (101, 102). However this hypothesis remains unproven, and other factors such as autoimmunity, genetics and β-cell recovery may contribute to the observed sex differences (37, 103).

##### 7.1.4.2 Age

Age at disease onset is a significant determinant of PR (20, 25, 35, 36, 41), but the relationship is not linear. This justifies dividing the study cohorts into three age groups (37). In the DPV registry, children aged 5–10 years and those aged ≥10 years had twice the odds of entering PR compared to those <5 years old (OR = 2.08; OR = 2.16, respectively, p <0.0001). Moreover, quantile regression revealed that a younger age (<5 years) was associated with a significantly shorter PR duration (by 3.2 months) versus the ≥10-year-old group (p <0.0001) (37). Zhong et al. reported a clear age gradient in PR frequency in their Chinese cohort (25). The proportion of children entering PR decreased progressively with younger age at onset: 87.5% among adolescents aged 13–18 years; 70.1% among those aged 7–12 years; only 46.5% among children aged <6 years. Multivariable analysis revealed that older age at onset was associated with a higher likelihood of PR (OR 12.57 for 7–12 years vs. ≤6 years; OR 3.40 for 13–18 years vs. ≤6 years). Older age was positively correlated with a longer PR duration (p = 0.001), independently of fasting C-peptide levels (25). Likewise, this age-dependent pattern was evident in the Hvidoere cohort: significantly fewer children under age 5 experienced PR vs. those aged 5–9.9 or 10–16 years (p <0.01) (20). In multivariate analyses, age at onset was a strong positive predictor of residual β-cell function (estimate = 0.09 per year, p <0.001). Mortensen et al. excluded age from the IDAA1c formula to preserve its clinical simplicity. Although stimulated C-peptide concentrations were highest among adolescents, PR rates according to the IDAA1c definition did not differ between the 5-9.9-year-old and ≥10 year-old groups, reflecting increased insulin resistance during puberty (20). In a cohort of 186 children with new-onset T1D, Pyziak et al. stratified patients by predicted pubertal status. They found that the likelihood of developing PR dropped 2.7-fold after entering puberty (OR = 0.37), though the association was no longer significant in multivariate analysis (OR = 0.85; p = 0.74) (29).

Reduced PR rates in early childhood may reflect accelerated β-cell destruction, delayed presentation/diagnosis, or distinct pathophysiological mechanisms in this age group. The second decline in PR occurrence observed during adolescence is likely related to the puberty-induced increase in insulin resistance. Age at diagnosis is a consistent predictor of PR occurrence and duration (35).

##### 7.1.4.3 Body mass index

BMI at diagnosis was positively correlated with the likelihood of PR in 167 pediatric patients (OR = 1.11; p = 0.032) (35), though this finding was not confirmed across cohorts (25, 37, 38, 41). In a new-onset T1D cohort of 99 children, Emet et al. described a significantly higher mean BMI-SDS increase in non-remitters versus remitters 6 months post-diagnosis (p = 0.04), though this increase was no longer significant after adjusting for pubertal status and sex (p = 0.09) (36). In a retrospective cohort of 119 T1D children conducted by Sokolowska-Gadoux et al., the initial BMI Z-score was positively associated with PR occurrence (28). Overweight and obese patients displayed a significantly higher probability of entering PR (78.9% and 100%, respectively, vs. overall PR incidence of 63% in the cohort). The authors stratified participants into three groups: non-remitters, remitters with short PR (<2 years), and remitters with long PR (≥ 2 years). The lowest BMI z-score was observed among non-remitters, intermediate values among PR <2 years group, and highest values among PR ≥ 2 years group. According to Pyziak et al., a higher BMI-SDS at diagnosis independently predicts PR (OR = 1.80; p = 0.0205) in multivariate analysis. However, this difference between remitters and non-remitters was no longer observed at 24-month follow-up (29). These findings were corroborated in a later study published in 2020 (56).

This association may be interpreted in light of the acceleration hypothesis. According to this concept, a higher BMI at diagnosis, reflecting increased insulin resistance, may exacerbate hyperglycemia and hasten the onset of overt clinical symptoms, leading to an earlier diagnosis of T1D. The prompt initiation of insulin therapy subsequently improves insulin sensitivity and reduces metabolic stress on the remaining β-cells. As a result, residual endogenous insulin secretion may temporarily become sufficient to contribute to glycemic control, favoring the occurrence of partial remission, despite the ongoing autoimmune destruction of β-cells (104, 105).

##### 7.1.4.4 Others

Authors have developed models incorporating clinical data for predicting PR. Marino et al. designed a clinical model predicting non remission with 73% power, based on the following criteria: bicarbonate <15mmol/L, age <5 years, female sex, and more than three diabetes-associated autoantibodies (38).

##### 7.1.4.5 Ethnic and socioeconomic factors

Socioeconomic and ethnic factors are studied as potential determinants of PR. A study involving a large group of New Zealand children found lower PR rates for Maori, Pacific (28.6%, p = 0.006), and other non-European ethnic backgrounds (28.8%, p = 0.03) versus New Zealand Europeans (50.4%) (41). Children from the most deprived areas were less likely to enter PR. Redondo et al. reported lower PR rates in African American children versus non-Hispanic white peers (42). Children from racial and ethnic minority groups tended to have lower residual β-cell function, as measured by IDAA1c, and clinical characteristics associated with greater long-term risk. IDAA1c and insulin dose requirements were higher for Hispanic children than for non-Hispanic whites. Even after adjusting for age, sex, and BMI, daily insulin needs were significantly higher in African American than white children. When corrected for known race-related differences in HbA1c values, the gap in HbA1c between African American and white patients remained significant at 6 and 12 months post-diagnosis (106). These findings highlight the need to establish population-specific criteria so as to avoid underestimating PR in certain ethnic groups (107).

##### 7.1.4.6 Physical activity

Regular physical activity (PA) plays a decisive role in PR occurrence and duration in new-onset T1D children. A study by Jamiołkowska-Sztabkowska et al. found that physically active patients underwent PR more often and for longer periods than their non-active peers, which led to improved metabolic control and reduced insulin requirements over two years (27). This aligns with previous literature on PA benefits for young T1D children (108–111). C-peptide remains the most reliable marker of residual β-cell function, though its interpretation in the PA context is complex. While absolute levels were similar across groups, physically active children more often maintained significant C-peptide secretion and exhibited better metabolic outcomes. This suggests that PA improves insulin sensitivity, changing the relationship between C-peptide secretion and β-cell function. Despite the clear therapeutic PA benefits, less than half of the cohort’s children met the recommended levels of PA.

#### 7.1.5 Biochemical parameters

##### 7.1.5.1 HbA1c

Remitters have better metabolic control during first years of disease, as shown by lower HbA1c levels. However, regarding HbA1c levels at diagnosis, contradictory data have been reported: some studies found no association with PR occurrence (23, 25, 27, 28, 35), while others identified a negative correlation. Among 242 T1D children, HbA1c levels measured at clinical onset negatively correlated with occurrence of PR (aOR 0.87, p = 0.03) (39). In this cohort, HbA1c was also a predictor of PR duration, as patients displaying HbA1c levels ≤ 6% after 3 months had longer PR duration (> 1 year) (39). Wong et al. reported that non-remitters had higher initial HbA1c level (p = 0.029), with an unadjusted relative risk of 1.12 (40). Comparing remitters to non-remitters in a cohort of 204 children, Marino et al. reported similar HbA1c levels at diagnosis between both groups and then significantly lower HbA1c levels in remitters compared to non-remitters from 3 months until 18 months of follow-up (38). Emet et al. and Pyziak et al. found a negative correlation between HbA1c levels at onset and PR occurrence (p = 0.04 and p = 0.0223 respectively) (29, 36). In the DPV cohort, higher HbA1c levels at diagnosis were associated with a lower odds of entering PR (OR = 0.85, p <0.001) and a shorter PR duration (- 0.5 months per 1% increase in HbA1c, p <0.001) (37). A higher HbA1c level at diagnosis likely reflects greater glucotoxicity and, consequently, a reduced functional β-cell reserve.

##### 7.1.5.2 Diabetic ketoacidosis

In several cohorts, DKA at diagnosis was associated with a lower probability of entering PR (35, 37, 39, 41). In the DPV cohort, the odds of PR were higher in children without DKA (OR 1.41; 95% CI 1.13-1.71) (37). In a cohort of 614 children with new-onset T1D, DKA was associated with lower odds of PR (aOR 0.54) after adjusting for sex, age at diagnosis, ethnicity, DKA, diabetes duration, BMI SDS at diagnosis, and socioeconomic status (41). In Passanisi et al., DKA at onset was more common among non-remitters than remitters (58.9% vs. 43.2%, p = 0.044). PR was more common with mild (venous pH <7.3 or bicarbonate <15mmol/L) than with moderate/severe DKA (p = 0.015) (35). Pecheur et al. reported a lower likelihood of PR for T1D children who presented with DKA at onset (adjusted OR 0.43, p = 0.018) (39).

According to ISPAD, DKA is defined by ketosis, hyperglycemia, and acidosis, with the latter specified as a venous pH <7.3 or bicarbonate level <15 mmol/L (112). Marino et al. observed a significant difference in serum bicarbonate at diagnosis between remitters and non-remitters, without a corresponding difference in pH levels <7.35 or DKA defined dichotomously. The authors suggested a bicarbonate threshold <15mmol/L may be more sensitive of acidosis and reduced residual β-cell function than pH values or conventional DKA criteria (38). Chobot et al. found no significant difference in DKA prevalence between remitters and non-remitters. However, pH at presentation was higher in those entering PR (7.33 ± 0.11 vs. 7.28 ± 0.18, p = 0.03) (44). According to Pyziak et al., remitters presented a significantly higher median pH at diagnosis versus non-remitters (7.38 vs. 7.33, p = 0.0096), but this association was no longer significant after multivariate adjustment (OR = 0.74; p = 0.89) (29).

Patients who present with DKA at onset of T1D exhibit severe insulin deficiency resulting from advanced pancreatic β-cell destruction. The metabolic stress induced by DKA further exacerbates this condition (39). In addition, DKA at onset is associated with poorer long-term glycemic control (113).

##### 7.1.5.3 Islet auto antibodies

Several studies investigating autoantibody status at diagnosis reported a negative correlation between high autoantibody titers and PR occurrence (37, 38) or duration (39); other cohorts failed to demonstrate any significant relationship (23, 25, 29, 36, 41, 44). While a slight association between ethnic background and specific autoantibodies has been described (42), most studies found no association between PR occurrence and any particular autoantibody at onset (23, 35). Some authors however suggest that the presence of multiple autoantibodies at diabetes onset reflects a more aggressive autoimmune process and a faster decline in endogenous insulin secretion during the first years following diagnosis (114).

There was a substantial heterogeneity across studies in the autoantibody types measured and detection methods used. Autoantibodies assessed were as follows: islet cell autoantibodies (ICA), insulin autoantibodies (IAA), glutamic acid decarboxylase 65 autoantibodies (GAD_65_A), insulinoma-associated protein 2 autoantibodies (IA-2A; also known as ICA512), and β-cell specific zinc transporter 8 autoantibodies (ZnT8A). Detection methods comprised enzyme-linked immunosorbent assay (ELISA), radio-binding assay (RBA), and immunofluorescence.

## 8 Other biomarkers: outside standard of care

### 8.1 Hormones

#### 8.1.1 Proinsulin

Kaas et al. found that proinsulin levels were significantly higher in remitters compared to non-remitters at 6 and 12 months. Measuring proinsulin at one month could predict remission status at six months (55). Proinsulin is usually considered a β-cell stress marker (115, 116). After T1D onset, the remaining β cells face an increased demand, which leads to physiological stress on the endoplasmic reticulum, misfolding of proinsulin and consequent release into the circulation (93). Kaas et al.’s study found a positive association between proinsulin and C-peptide, and an inverse correlation between proinsulin and IDAA1c (55). These authors interpreted higher proinsulin concentrations in remitters as reflecting a residual pool of active β-cells. Given that proinsulin is a major autoantigen in T1D, they speculated that increased exposure might favor regulatory T-cell-mediated tolerance. Supporting this view, clinical studies in children have shown that administration of proinsulin peptides can induce antigen-specific immune modulation, with increased Treg frequencies and interleukin 10 (IL-10) production, correlating with better preservation of β-cell function (117). Mechanistic work has demonstrated that central tolerance to proinsulin is regulated by its thymic expression, and that a breakdown of this process predisposes to β-cell autoimmunity (118). Other authors have proposed that enhanced proinsulin secretion could generate neoepitopes, thereby potentiating autoreactive immune responses (119). Clarifying whether elevated proinsulin primarily promotes immune regulation or fuels autoimmunity requires dedicated mechanistic studies.

These findings contrast with those of Sabek et al., who found no association between proinsulin or the proinsulin/C-peptide ratio and PR in children with recent-onset T1D (54). This discrepancy is likely explained by differences in study design and patient populations. Whereas Kaas et al. assessed meal-stimulated proinsulin longitudinally at several time points during the first year after diagnosis, Sabek et al. relied on a single random proinsulin measurement obtained approximately seven weeks after diagnosis, which may less accurately capture residual β-cell secretory capacity. In addition, the Sabek cohort included more children presenting with DKA, which markedly increases β-cell stress and could obscure any association between proinsulin levels and PR status. Their population was also smaller and more heterogeneous, with wide variability in age and BMI; after adjustment for these factors, proinsulin was no longer associated with PR. Taken together, these differences highlight the context-dependent nature of proinsulin as a biomarker: depending on timing and metabolic stress, elevated proinsulin may reflect either endoplasmic reticulum stress and impaired prohormone processing or, as observed in longitudinal stimulated assessments, the persistence of functionally active β cells.

In T2D, elevated proinsulin is a well-established marker of β-cell stress and defective prohormone processing, arising both from increased secretory demand due to insulin resistance and intrinsic β-cell dysfunction, as evidenced by the disproportionate hyperproinsulinemia observed in patients with diabetes compared to obese controls (120). Experimental models of acute β-cell workload, like partial pancreatectomy, have further shown that proinsulin elevation primarily reflects functional secretory dysfunction, rather than reduced β-cell mass (121). Treatments that alleviate insulin resistance like insulin or incretin-based therapies improve metabolic control and decrease circulating proinsulin levels (122, 123). Current data from T1D and T2D studies indicate that proinsulin may reflect β-cell stress and residual secretory capacity; its interpretation likely depends on disease stage, metabolic demand and immune context.

#### 8.1.2 Bone turnover markers

Abundant evidence has revealed that T1D patients exhibit alterations in bone metabolism (124). Pediatric T1D patients have lower bone mineral density (BMD) than healthy controls, which increases their fracture risk (125). Disturbances in bone turnover markers were reported in children with T1D, emerging as early as the clinical onset (126). We know insulin promotes bone formation by stimulating osteoblastic activity and the synthesis of matrix proteins such as osteocalcin (OC) and osteoprotegerin (OPG). In states of insulin deficiency, a reduction in bone formation and an alteration of bone microarchitecture are observed (127). T1D also promotes the accumulation of advanced glycation end products on collagen. This non-enzymatic glycation increases the stiffness of collagen fibers through abnormal cross-linking, making bone more brittle and less resistant to fractures (128).

In a cohort of 99 children, Madsen et al. reported decreased osteocalcin and procollagen type 1 amino-terminal propeptide (P1NP) levels (markers of bone formation), and increased c-terminal crosslinked telopeptide of type 1 collagen (CTX, a marker of bone resorption) compared to reference population during the first year after T1D onset (24). No differences in these markers were observed between patients in and out of PR. However, the longitudinal increase in CTX was negatively associated with the rise in IDAA1c, independently of age and C-peptide decline. The slower increase in IDAA1c with increasing bone resorption led to consider bone resorption as a compensatory mechanism enhancing insulin sensitivity.

Sabek et al. highlighted undercarboxylated osteocalcin (uOC), the biologically active fraction of osteocalcin, which modulates energy metabolism and insulin action (54). In their study, children with DKA at onset demonstrated a positive correlation between the ratio of undercarboxylated to carboxylated osteocalcin (uOC/cOC) and C-peptide levels (p = 0.03), and a negative correlation between uOC and proinsulin/C-peptide ratio (p = 0.03). In a previous report, uOC and uOC/cOC ratios were inversely correlated with HbA1C in children with recent T1D onset (129). Therefore, osteocalcin may provide complementary insights into residual β-cell function and metabolic status in new-onset T1D.

Szymańska et al. assessed OC, OPG, soluble receptor activator of NF-κB ligand (s-RANKL), and urinary deoxypyridinoline (DPD) at diagnosis and after seven months in 100 children with newly diagnosed T1D (30). OC was reduced and OPG elevated at onset compared to controls, while -RANKL and DPD increased during follow-up. OC at onset correlated positively with fasting and stimulated C-peptide and with blood pH, and negatively with HbA1c. This indicates a link between better metabolic status, preserved β-cell function, and bone formation activity. Contrarily, OPG correlated negatively with C-peptide and pH and positively with HbA1c. Children in PR at seven months had lower urinary DPD levels, suggesting reduced bone resorption and higher total body BMD Z-scores. The authors concluded that bone metabolism abnormalities are evident at T1D onset and are dynamically modulated by metabolic control.

#### 8.1.3 Glucagon and incretins

In a cohort of 275 newly diagnosed juvenile T1D patients, remitters had lower postprandial (90 minutes) levels of glucagon (10.0 vs. 12.0 pmol/L at 6 months, p <0.0001; 11.0 vs. 13.0 pmol/L at 12 months, p = 0.02) and GLP-1 (16 vs. 22 pmol/L at 6 months, p = 0.0001; 19 vs. 24 pmol/L at 12 months, p = 0.002) than non-remitters. Measuring postprandial glucagon and GLP-1 at 1 month could predict remission status at 12 and 6 months, as shown by logistic regression analyses adjusted for age, gender, and stimulated blood glucose (55). Lower postprandial GLP-1 levels in remitters can be explained by the strong correlation between GLP-1 secretion and postprandial glycemia, which is reduced during PR. Lower glucagon responses during PR suggest that glucagon secretion by α cells is inhibited in an islet environment where β cells still secrete insulin upon glucose stimulation, as seen in PR. To our knowledge, no pediatric studies linking lower postprandial glucagon to PR have incorporated healthy controls. Reference values must thus be inferred from pediatric case-control MMTT studies that are not stratified by remission status (130).

### 8.2 Variations in immune cell subsets

#### 8.2.1 Innate immunity

##### 8.2.1.1 Neutrophils

In a cohort of 28 pediatric patients with new-onset T1D and 28 healthy age- and gender-matched controls, Fitas et al. (50) found that T1D children at onset had lower neutrophil counts than healthy controls. The counts increased during PR, and rose further in established disease, reaching levels comparable to those of healthy controls. The initial reduction in neutrophils at diagnosis was attributed to IL-17–mediated recruitment of these cells to the pancreas, accompanying the active phase of β-cell destruction (131). Their subsequent increase in the periphery during PR may reflect a lower migratory drive towards pancreatic tissue and enhanced medullary neutrophil differentiation promoted by Th17 immunity (132). Consistently, IL-17-producing cells followed a similar temporal pattern, reinforcing the view that the Th17-neutrophil axis plays a primary role in T1D immunopathology. Neutrophils were broadly defined as CD45^+^/SSC^high^ cells by flow cytometry, without distinction of functional subpopulations. Gómez-Muñoz et al. (51) observed lower absolute counts of CD16^+^ neutrophils at 8 and 12 months post-diagnosis in non-remitters compared to remitters. Beyond tissue retention, they suggested that impaired neutrophil maturation or proteolytic cleavage of FcγRIIIB may underlie the altered phenotype observed in non-remitters.

In a cohort of 85 children with new-onset T1D, Klocperk et al. reported a progressive decrease in granulocytes in peripheral blood during first year after disease onset, along with a gradual decrease in neutrophils proportion (p=0.0016). While most variations in innate immune cells were subtle, the percentage of neutrophils dropped below healthy levels in some patients at 6- and 12-months post-diagnosis (53). Discrepancies across studies likely reflect differences in design, stratification, and readouts. Fitas et al. sampled patients at clinically defined phases, whereas Klocperk et al. assessed fixed timepoints (0, 6, 12 months), irrespective of remission status. They reported a decline in granulocytes and a lower neutrophil percentage among leukocytes. These findings could be diluted by the inclusion of non-remitters and by using proportions rather than absolute counts. Gómez-Muñoz et al. observed lower CD16^+^ neutrophils at 8–12 months in non-remitters versus remitters, pointing to PR-dependent neutrophil dynamics. This suggests that neutrophil trajectories rise with PR when analyses are aligned to clinical phases and absolute counts.

##### 8.2.1.2 Natural killer cells

Fitas et al. (50) reported a reduction in circulating NK cells at T1D onset that persisted as the disease progressed. Gómez-Muñoz et al. (49) confirmed reduced NK cell counts during the first year post-diagnosis. This reduction, particularly evident in the CD56^dim^ subset, was also observed in other autoimmune diseases (52), reflecting NK cell migration to the pancreas and draining lymph nodes. Reduced NK cells in peripheral blood over time may reflect functional hypo-responsiveness, a hypothesis supported by the shedding of CD16, a key mediator of ADCC, documented in T1D patients after diagnosis (49).

NK cells subsets exhibit distinct dynamics during PR. CD56^bright^CD16^-^ NK cells, corresponding to regulatory NK cells (NKregs), are decreased in the peripheral blood of children with new-onset T1D (52), possibly due to homing to lymphoid tissues. However, their numbers tend to increase during the first few months post-diagnosis, which coincides with the peak PR occurrence (33, 49). This expansion suggests a state of immunoregulation, as NKregs produce anti-inflammatory cytokines like IL-10 and can suppress autoreactive T cells or eliminate overstimulated immune cells. Their exhaustion may contribute to PR termination. Intermediate CD56^bright^CD16^+^ NK cells are reduced during PR, suggesting that PR may restrict NKregs transition into intermediate subsets, a process usually driven by T-cell-derived cytokines and direct cell–cell interactions.

Expansion of a CD56^dim^CD16^–^ NK subset was reported later in the disease course, particularly in non-remitters, where their higher numbers may indicate increased trafficking from the bone marrow to the pancreas, promoting cytotoxic β-cell destruction. CD16 expression on total NK and effector NK subsets is reduced after diagnosis, reflecting exhaustion from repeated immune interactions. This reduction is especially marked in non-remitters at eight months versus onset and 12 months. CD16 expression by 12 months may indicate restored cytotoxic function (49).

##### 8.2.1.3 Dendritic cells

Dendritic cells influence the balance between tolerance and autoimmunity (133). Gomez-Muñoz et al. found that remitters had a lower percentage of dendritic cells (DCs) than non-remitters (51). This may be explained by the translocation of CCR2^+^ dendritic cells (DCs) from the circulation to target tissues, prompted by the release of CCL2 from inflamed islets (134). Reduced hyperglycemia during PR could contribute to the recovery of DCs’ immunosuppressive properties. Bechi Genzano et al. demonstrated an increase in plasmacytoid DCs in newly diagnosed T1D, not seen in other autoimmune diseases. A marked imbalance in peripheral dendritic cells, with elevated pDCs and reduced mDCs compared to controls, is observed at diabetes diagnosis (52). Klocperk et al. found a marked increase in pDCs in circulation 12 months after disease onset (adjusted p <0.0001) (53). This increase may be due to egress from the pancreas to peripheral blood or increased differentiation from precursors.

##### 8.2.1.4 Monocytes

Monocytes are precursors of antigen-presenting cells. A low concentration of circulating monocytes was associated with T1D onset and progression in the second year. Their decrease in peripheral blood two years after disease onset, when PR is terminated, suggests an active extravasation to target tissues as the autoimmune response becomes chronic (33).

#### 8.2.2 Adaptative immunity

##### 8.2.2.1 B cells/Bregs

In late-stage T1D, B lymphocytes are the most abundant immune population in pancreatic islets after CD8^+^ T cells, and their frequency inversely correlates with the amount of detectable insulin. Fitas et al. found that the absolute count of circulating B-cells was lowest during PR (50). In a longitudinal follow-up of 17 pediatric patients with new-onset T1D, Gomez-Munoz et al. found in remitters elevated levels of transitional Type 1 (T1, or transitional high) B lymphocytes at disease onset and during the first year (51). Patients at 12 months presented a higher T1/T2 ratio compared to onset of the disease (51).

Villalba et al. described an increase in the total B transitional subset (T1 and T2) during the first year after T1D diagnosis, followed by a decline in T1 during the second year (33). The transitional low subset (T2) peaked during the first year and decreased thereafter (33).

During the first year, the absolute numbers of regulatory B cells (Bregs) increased, suggesting an early attempt to restore self-tolerance (33). Bregs expansion was particularly evident at 1-year follow-up in remitters (51). Since both T1 transitional B cells and Bregs exert immunosuppressive functions by limiting effector T-cell proliferation, a higher T1/T2 ratio together with increased Breg counts found during PR may reflect the immunoregulatory mechanisms underlying this period.

##### 8.2.2.2 T cells/Tregs

Th17 and Tc17 cells are pro-inflammatory CD4^+^ and CD8^+^ T lymphocytes (74). They have been involved in T1D (135) and various autoimmune diseases (136). Fitas et al. found a decrease in Th17 and Tc17 cells at disease onset, then a recovery at PR, and a significant increase as disease progresses (50). Recruitment to target organs and draining lymph nodes may cause decreased numbers of circulating IL-17 producing cells at onset. Villalba et al. found an increased percentage in Th17 cells during the first year, when PR usually occurs, compared to second year (33).

At disease onset, EM (effector memory) and EMRA (terminally differentiated) T cells both in the CD4^+^ and the CD8^+^ T cell subsets decrease in peripheral blood (33). The recruitment of autoreactive T lymphocytes through the expression of the pro-inflammatory chemokine, CXCL10, in islets is one possible explanation (137). B cells from patients diagnosed with T1D exhibit heightened HLA class I expression (138), which increases the presentation of autoantigens to CD8^+^ T cells with an antigen-experienced phenotype (CD45RA^-^). This was seen in the islets (139). During PR, remitters show an increased percentage of CD4^+^ and CD8^+^ T_ERMA_ lymphocytes and EM CD4^+^ T lymphocytes, which could reflect their decreased activity in the pancreas (51). Interestingly, a reduction of EM CD8^+^ T cells is a shared marker with other autoimmune diseases (52).

Tregs are thought to be impaired at T1D onset due to preclinical activation (50). Villalba et al. found a decrease in memory regulatory T-cells (mTregs) at T1D onset, while activated regulatory T cells (aTregs) increased during the first year when PR occurs (33). Among pediatric patients with newly diagnosed T1D, Moya et al. found that the relative aTregs frequency at baseline correlated with PR length (21). Klocperk et al. reported a significant increase in total Tregs during the first year, with Tregs numbers remaining within the healthy range of peripheral Tregs (*i.e*., between 2 and 8% of CD4^+^T cells) (53). This could reflect a tolerogenic response to pancreatic inflammation, partly driven by elevated pDCs promoting Treg differentiation (140, 141). Starosz et al. reported that children with lower HbA1c values and lower insulin dose requirements at disease onset had higher absolute Tregs numbers (32). Patients with initially higher Treg counts had reduced insulin needs. A higher Treg/Th17 cell ratio was linked to a higher chance of PR. This association faded after six months, along with the decline in PR frequency and rise in insulin requirements (32). There are conflicting data on Tregs due to different methods of identifying these cells (51). Fitas et al. (50) described a significant decline in Tregs after PR, while Gomez-Muñoz et al. (51) found a decrease in the percentage of CD4^+^ CD25^+^ CD127^low^ (Tregs) and memory Tregs during PR versus no PR, with a maintained difference after first year post-diagnostic for Tregs.

The influence of Tregs on the pathophysiology of T1D and PR warrants further investigation. Studies have shown that a higher Tregs/Teffector cell ratio can lead to PR (142). Fitas et al. showed an increase in Th17/Tregs ratio after PR and set out that the classic Th1/Th2 paradigm might not be sufficient to understand immunopathogenic events in T1D (50). Detecting an exhaustion-like profile on CD8+ T cells could help identify patients with slower disease progression (143). Rectification of hyperglycemia following insulin therapy initiation could modulate Tregs’ functionality (6).

A study of pediatric patients with newly diagnosed T1D by Moya et al. revealed that the frequency of CD45R0^+^ memory cells and CD25^+^ CD127^high^ cells at diagnosis was strongly correlated with PR length (21). CD25^+^CD127^high^ cells (which are neither Treg nor Tr1 cells) represented mainly central and transitional memory CD4^+^ cells. Their frequency was the strongest independent predictor of PR duration, especially when combined with baseline HbA1c, IDAA1c or C-peptide. According to the authors, the CD25^+^CD127^high^ contributes to immune balance and slower disease progression during the PR phase (21).

#### 8.2.3 Cytokines

Cytokines are known to play an important role in T1D pathogenesis by controlling innate and adaptative immune responses, thereby contributing to the initiation and amplification of β cell autoimmunity. Pro-inflammatory cytokines like IFN-α, IFN-γ, IL-1β and TNF-α, are produced by immune cells infiltrating pancreatic islets and impair β cell function and survival, ultimately promoting the development of T1D (144).

Fitas et al. identified two cytokine response profiles in their remitters cohort. Low responders (LR) had no detectable anti-inflammatory cytokines (IL-4, IL-10, or IL-13) and no pro-inflammatory TNF-α. High responders (HR) had at least one of these cytokines detected (50). The LR profile showed higher fasting C-peptide levels at onset and longer PR periods. HR presented higher levels of other cytokines (IL2, IL-6, IFN-γ, and MCP-1) at onset, PR (IL2, IL-17A, and IFN-γ), and after disease establishment (IL1-β, IL2, IL-6, IL-17A, IFN-γ and MCP-1). The anti-inflammatory cytokines were expressed with the pro-inflammatory cytokine TNF-α, suggesting a reactive response to counteract its toxicity. Evidence suggests that cytokine activity depends on the timing of disease course, concentration, and surrounding cytokine milieu (145).

Overgaard et al. examined prospectively the association between systemic cytokine levels and residual β-cell function in 63 children/adolescents with newly diagnosed T1D (31). TNF-α, IL-2 and IL-6 concentrations were inversely correlated with stimulated C-peptide concentrations at 6 months. This shows a link between systemic inflammation and β-cell decline. Higher baseline TNF-α and IL-10 levels independently predicted lower stimulated C-peptide concentrations at 6 months. The authors identified TNF-α as the most consistent correlate of β-cell dysfunction and a potential prognostic biomarker of PR and disease progression (31).

A study of 60 pediatric patients with newly diagnosed T1D by Starosz et al. found that a high IL-10 levels at diagnosis indicated severe inflammation, whereas a low IL-10/IL-17 ratio indicated PR (32). In patients with high IL-10 concentrations, this cytokine was positively correlated with both HbA1c (p <0.01) and insulin requirements (p <0.05). Elevated IL-10 levels suggest a compensatory but insufficient anti-inflammatory response linked to poorer metabolic control. IL-10’s role is ambiguous, as IL-10 can limit excessive immune activation, but also contribute to chronic inflammation and disease progression. In patients with lower initial IL-17 concentrations, IL-17 plasma levels negatively correlated with HbA1c at onset and with C-peptide at 24 months (both p <0.05). This association diminished when IL-10/IL-17 ratios were considered. The immune balance between regulatory and proinflammatory pathways likely plays the predominant role in controlling autoimmune diabetes (32).

A study of 22 newly diagnosed T1D patients by Villalba et al. found a decline in circulating levels of transforming growth factor-beta (TGF-β) among remitters at 6 and 12 months after disease onset (33), though circulating TGF-β levels at onset were similar for remitters and non-remitters. This suggests that the disease’s progression, not its severity at DT1 onset, is the distinguishing factor. No significant differences in TGF-β levels were found between remitters and non-remitters at 6 and 12 months after disease onset. TGF-β levels could thus be used to monitor PR in individual patients, but they lack the capacity to predict PR occurrence (33).

Fetuin-A, a circulating liver-derived glycoprotein, inhibits the tyrosine-kinase activity of the insulin receptor, conferring a pro-insulin-resistant effect and serving as a marker of peripheral insulin resistance. IL-8, a chemokine produced by adipocytes and tissue macrophages, contributes to adipose-tissue inflammation and can induce insulin resistance by disrupting insulin signaling in human adipocytes. In a cohort of 134 children with newly diagnosed T1D, Pyziak-Skupien et al. found that fetuin-A and IL-8 concentrations at diagnosis were significantly higher in remitters versus non-remitters (56). Fetuin-A levels were also higher in children without GADA and ICA autoantibodies, suggesting interplay between insulin resistance and the autoimmune process. However, in multivariate logistic regression, only BMI SDS independently predicted PR (OR 2.305). The authors hypothesized that insulin resistance overlapping with initiated, yet clinically silent, autoimmune β-cell destruction could lead to earlier hyperglycemia with less intense β-cell loss. Patients with better preserved residual β-cell function at diagnosis could temporarily recover with insulin therapy, enabling PR (56).

Gomez-Muñoz et al. reported no significant differences in the analyzed cytokines (IL-2, IL-6, TGF-β1, IL-17A, and IL-10) between remitters and non-remitters (51). The authors contended that using cytokines as markers of T1D progression lacks sufficient power to accurately differentiate PR.

### 8.3 HLA genotyping

In a cohort of Brazilian children and adolescents with T1D, Camilo et al. found that PR occurred more frequently in children bearing the HLA class II DRB1*03-DQB1*0201 haplotype (23). Homozygotes for this haplotype had a significantly lower prevalence of autoantibodies to IA-2A (protein tyrosine phosphatase or ICA512) at diabetes onset compared to heterozygotes. However, there was no significant difference in IA2 antibody titer reduction between remitters and non-remitters. Homozygous patients with HLA class II haplotypes have decreased autoantibody synthesis, which may be due to reduced diversity of alleles recognizing islet antigens. Fewer antigens presented to autoreactive CD4^+^ T lymphocytes could lead to decreased antibody production.

Among 63 patients with childhood-onset T1D, Chen et al. reported that children with the DR9/DR9 genotype had a lower PR rate (45.4% vs. 70.2%, p = 0.022) (26). There was a negative relationship between DR9/DR9 and PR in children (OR = 0.196, p = 0.004). DR9/DR9 was more frequent in adult-onset T1D than in childhood-onset (p = 0.006). DR9/DR9 impacted PR quality, as assessed by peak C-peptide levels, likelihood of achieving peak C-peptide ≥400pmol/L, and time from diagnosis to reach peak C-peptide level. However, it did not have such an effect in childhood-onset T1D. When not grouped by age, DR9/DR9 carriers displayed a specific pattern: older age of onset, higher percentage of adults, and lower prevalence of positive ZnT8A. However, no relationship between DR9/DR9 and ZnT8A was found in children (26). According to the authors, acute β-cell destruction in T1D is likely associated with a lower probability of entering PR. DR9/DR9 is a high-risk genotype in Asian populations (146), as evidenced by more acute β-cell function loss. Previous Japanese research has shown that DR9/DR9 is more prevalent in patients with acute-onset T1D than in those with slowly progressive disease (147). This correlates with a faster progression to complete β-cell failure (148) and greater insulin dependency (149). According to the authors, early intensive insulin therapy could increase the likelihood of PR in DR9/DR9 carriers, since glycemic normalization and immune modulation are key mechanisms underlying PR.

### 8.4 MicroRNAs

MicroRNAs are small, non-coding RNAs that regulate gene expression post-transcriptionally through various mechanisms. Their role as serum biomarkers has been demonstrated in many diseases, including autoimmune disorders (150). Altered expression of specific miRNAs has been associated with impaired regulation of T-lymphocyte development, differentiation, and function (151). Circulating miRNA profiles vary among patients with recently diagnosed T1D, long-standing T1D, and healthy controls, which suggests that peripheral miRNA signatures reflect different disease stages (60).

Samandari et al. (2017) found that higher hsa-miR-197-3p levels at 3 months post-diagnosis were linked to a sixfold increase in residual β-cell function, as measured by stimulated C-peptide at 12 months (59). Target genes of hsa-miR-197-3p, including BME and PMAIP1, are involved in β-cell apoptosis. The levels of hsa-miR-375, hsa-miR-301a-3p, hsa-miR-24-3p, hsa-miR-194-5p, and hsa-miR-146a-5p at 3 months predicted stimulated C-peptide, IDAA1c, and HbA1c at 6 and 12 months. These markers have been associated with β-cell growth, development, differentiation, and function (152, 153). However, no predictive miRNA profile was identified at 1-month post-diagnosis. In 2018, Samandari et al. reported dynamic changes in eight circulating miRNAs during the first five years post-diagnosis (60). These miRNAs were associated with pancreatic autoantibodies and immunomodulatory cytokines. This suggests that circulating miRNA profiles reflect ongoing immune processes during PR. They may help define the optimal window for immunotherapies.

In 2023, Gómez-Muñoz et al. identified a miRNA signature specifically associated with PR (58). The authors found that remitters had 12 upregulated miRNAs, five of which belonged to the MIR-17 family. This family has been linked to immunomodulation and β-cell apoptosis/regeneration. Of the upregulated miRNAs, miR-30d-5p was the most significant and differentially expressed during PR, making it a promising candidate biomarker of PR. This glucose-regulated microRNA is associated with immune system function, stress response, cell death, insulin receptor signaling, insulin production, and the preservation of β-cell function (154). An experimental model of T1D (non-obese diabetic mice) demonstrated the immunoregulatory role of miR-30d-5p. Its inhibition *in vivo* (non remission scenario) led to increased CD4^+^ CD25^+^ FoxP3^+^ Tregs in pancreatic lymph nodes, decreased PD-1 expression on splenocytes, and a slight increase in insulitis (58).

### 8.5 Proteomics

Omics technologies are essential for understanding the mechanisms and biomarkers of diabetes (9, 155). Proteomics approaches have enabled research to identify proteins associated with diabetes progression from presymptomatic to clinical stage (156, 157) and serum signatures that distinguish T1D and T2D (158, 159). In the DIATAG cohort, we used mass spectrometry to identify candidate PR biomarkers (57). Considering PR as a continuous phenomenon enabled our group to detect differentially expressed plasmatic proteins at T1D diagnosis (47).

Peptides from TUBA4A, TUBB1, TUBB4B, YWHAZ, UNC13D, and CRKL showed strong correlation with the IDAA1c score (R ≥0.68, p <0.05). C-peptide estimates at 3 months post-diagnosis significantly correlated with TAOK1, SKAP2, WDR44, and UNC13D. Using a qualitative approach, we classified potential biomarkers in six diabetes-related clusters: insulin secretion, cellular stress, inflammation markers, lipid metabolism, muscle, and micro and macrovascular complications. When downregulated, Src-kinase associated protein 2 (SKAP2) increases β-cell sensitivity to cytokines, and when upregulated, it increases macrophage activity (160, 161). Crk-like protein (CRKL) overexpression is linked to autoimmune diseases in humans (162). Higher circulating YWHAZ levels could predict dysglycemia. Tubulins are linked to platelet dysfunction, inflammation, and cancer. Their origin is unclear, but they seem to be released in response to global metabolic stress and inflammation.

## 9 Discussion

In this review, we attempted to provide a relevant overview of the markers influencing the occurrence and duration of PR in pediatric T1D. The clinical parameters and biomarkers studied in this review were selected to reflect both the state of research in understanding the pathophysiological mechanisms of T1D and PR, and the clinical approaches that enable targeted patient management. To our knowledge, no studies have demonstrated a significant association between PR in pediatric T1D and findings from genome-wide association studies, genetic microarrays or genetic risk score. We used the IDAA1c score (and equivalent definitions: stimulated C-peptide ≥300 pmol/L or HbA1c <7% and <0.5 IU/kg BW/day), widely accepted in the literature, to define PR. The review is restricted to children/adolescents for several reasons. The peak age for diagnosing T1D is between 10 and 14 years (68, 163), with approximately 40% of T1D patients diagnosed before adulthood (68, 164). Furthermore, improving PR identification is crucial for patient quality of life, especially in pediatrics. Early PR detection could enhance the effectiveness of immunotherapies aimed at prolonging PR duration and thereby delay the “second hit” to a stage when patients are more mature and better able to manage their disease.

The risk of bias assessment indicated a moderate level for the 39 studies included. The main limitations identified are the frequent absence of multiple imputation, insufficient sensitivity analysis regarding attrition, and incomplete or heterogeneous consideration of confounding factors between studies. Nevertheless, the majority of studies used adequate statistical approaches and took into account clinically relevant confounding factors at the time of T1D diagnosis, including age, sex, initial DKA, autoantibodies, HbA1c, and BMI. Observational studies are well suited to explore a dynamic phenomenon such as PR and to generate hypotheses and candidate biomarkers. Their limited capacity for causal inference and susceptibility to bias were mitigated by systematically documenting methodological heterogeneity.

In this review, we show that CGM metrics reliably indicate PR. The GTAA1c score provides additional value to the IDAA1c criterion, specifically in certain subgroups. The TIR_70-180_, TIT_63-140_, et TAR_>180_ parameters, as well as the PHH parameters, are useful to distinguish between remitters and non-remitters. We identified four glucotypes, each associated with distinct clinical profiles and CGM metrics. This led us to characterize specific signatures of glycemic variability over 24 hours and refine the PR definition: in particular, patients with a borderline IDAA1c score can be stratified into glucotype 2 or 3. The gradual increase in glycemic variability observed between glucotypes was confirmed by the integration of PHH parameters, and the identification of specific thresholds for each of these parameters allows patients classification into clinically relevant groups, which can guide follow-up and therapeutic management.

Age and severity of the condition, reflected by the presence of DKA, HbA1c level and BMI at T1D onset, appear to be the most decisive factors in the progression of the disease. This observation should motivate the design of clinical studies that stratify patients more precisely (17), for example according to age. This approach would help to fill the current knowledge gap regarding the specificity of T1D diagnosed in patients under 5 years of age (165). Boys appear to be more likely to experience PR, but this observation is not confirmed in all cohorts. Studies examining the impact of gender on PR would benefit from systematically assessing pubertal status in order to observe differences in pubertal hormone coverage between the two sexes for the age groups observed.

Our work emphasizes the important role of educating young patients in managing their diabetes. We explain that physical activity, insulin dosage and food intake are the main determinants of glycemic variability. These factors can be effectively addressed by teaching children good dietary habits. The lower frequency of PR observed by Chiavaroli et al. among patients from socioeconomically disadvantaged backgrounds could be partly explained by more difficult access to healthcare and lack of targeted support.

Studies of immune cell subsets and hormones in relation to PR offer insights into T1D progression. Proinsulin is usually seen as a marker of stress and beta cell dysfunction but could also reflect functional β-cell reserve. Some suggest proinsulin may promote a certain degree of immune tolerance. This hypothesis has led to the development of a phase 1 clinical trial testing an antigen-specific immunotherapy consisting of nanoparticles loaded with human proinsulin peptide (166).

Bone turnover markers are exploratory biomarkers of beta cell function, but not directly of PR, as no association between these markers and the IDAA1c score has been demonstrated. Some authors report associations with metabolic markers such as HbA1C, BMI, DKA or pH at onset; or with β-cell function markers. Notably, in Sabek et al.’s study, only patients with DKA at onset show a significant but transient correlation between C-peptide and uOC/OC ratio, suggesting that acidosis and metabolic stress promote bone-pancreas coupling that does not appear in patients in better metabolic condition at clinical initiation.

**Figure 4:**
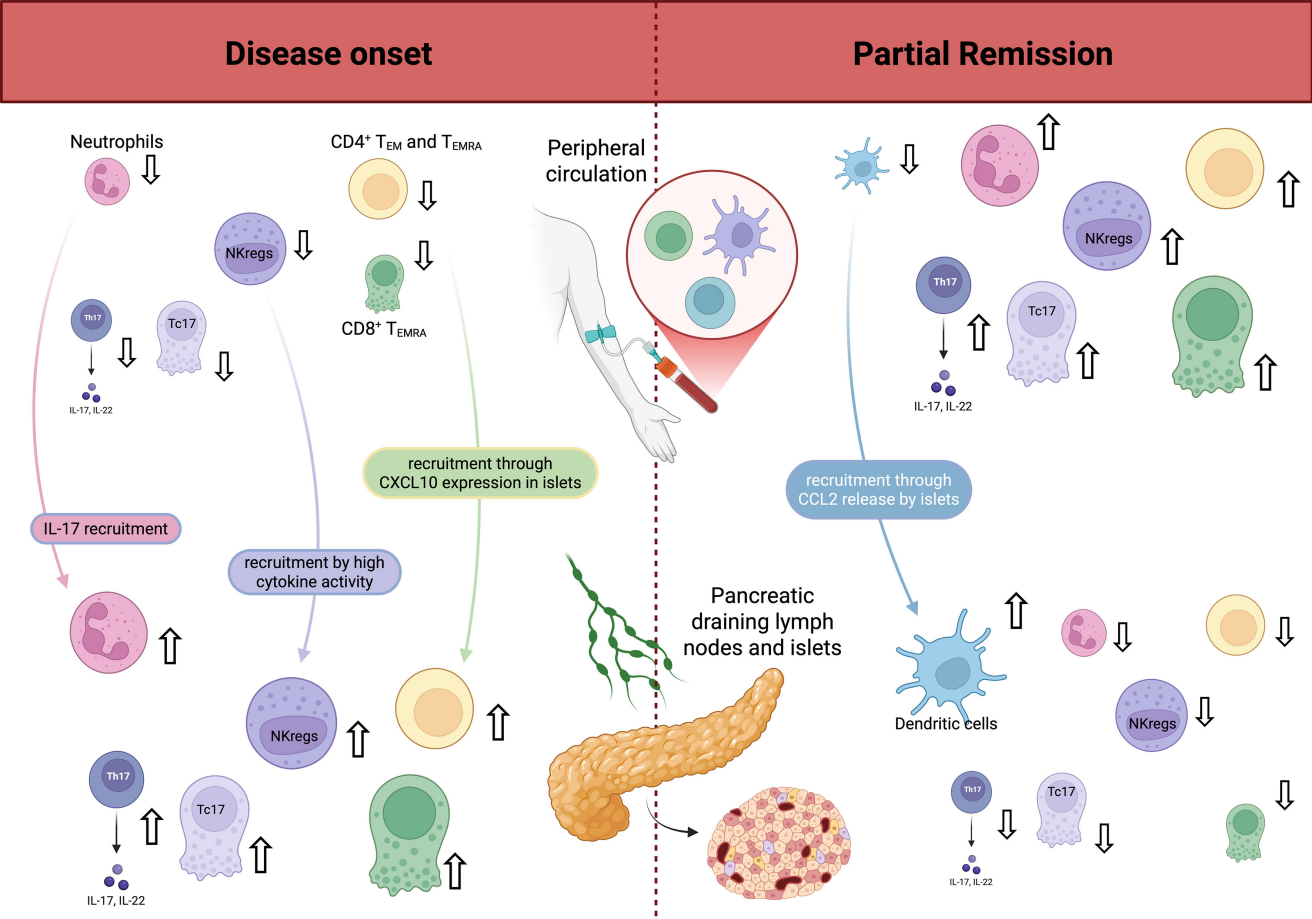
Graphical representation of immune cell dynamics associated with clinical disease onset and partial remission in pediatric patients with type 1 diabetes. Created with BioRender.com.

PR is a state of immunoregulation. We have compiled eight studies reporting peripheral changes in immune subpopulations during T1D progression. These studies suggest that certain effector cells - neutrophils, Th17 and Tc17, NKregs CD56^bright^CD16^-^, CD4^+^ CD8^+^ T_EMRA_, EM CD4^+^ T cells - migrate less towards the pancreas during PR, thereby increasing their abundance in the periphery. In contrast, regulatory cells such as dendritic cells could migrate more actively to the pancreas during PR, with a concomitant decrease in the blood (**Figure 4**). Migration of immune cells to the pancreas in the pre- and post-clinical stages of type 1 diabetes is well documented by several mechanistic studies in humans (167–171). However, to our knowledge, no study has yet specifically examined the dynamics of this migration or the peripheral redistribution of immune cells during the PR phase. Single-cell RNA sequencing, by revealing contrasts in phenotype and function between circulating lymphocytes and lymphocytes present in the pancreatic microenvironment (172), could clarify these mechanisms. Adult T1D studies support PR as an immunoregulatory state. Li et al. described restored PD-1/PD-L1 expression on T lymphocytes during PR (173) and Tang et al. demonstrated a modulation of intracellular carbohydrate metabolism in lymphocytes during this period (10).

This review summarizes six studies examining cytokines in relation to PR. The available data highlight that cytokine effects must be interpreted within the broader cytokine milieu. IL-10 exemplifies this complexity, as its role may be pro- or anti-inflammatory depending on the biological context. The study by Pyziak-Skupien et al. further underscores the importance of considering both metabolic and autoimmune status at diagnosis when interpreting fetuin-A and IL-8 levels. In addition, elevated TNF-α levels at diagnosis are associated with a decline in β-cell function. Collectively, these studies indicate that cytokine measurements have limited ability to discriminate remitters from non-remitters. Their value lies more in a dynamic approach that facilitates an individualized disease progression assessment.

PR appears to be more likely in young people carrying the HLA class II DRB1*03-DQB1*0201 haplotype and less likely in those homozygous for DR9 (DRB1*0901-DQB1*0303). These haplotypes do not seem to be linked to any particular diabetes-related autoantibodies in children. DR9/DR9, previously identified as a high-risk genotype for diabetes in Asian populations, is absent in several southern European populations and present at low frequency in other European regions (146).

MicroRNAs are promising circulating biomarkers for characterizing PR in T1D, but still of limited clinical use. The work of Samandari et al. shows that certain miRNAs measured after diagnosis, particularly hsa-miR-197-3p and several miRNAs involved in β-cell growth and survival, can predict changes in residual β-cell function in the following year. Other evolving profiles observed over the first five years of the disease seem to reflect immune dynamics rather than insulin secretion. More recently, the identification of a miRNA signature specific to remitters, dominated by the MIR-17 family and including miR-30d-5p as a particularly discriminating biomarker, reinforces the idea that certain miRNAs are closely linked to the immunomodulatory mechanisms known to accompany remission. Preclinical data supporting the immunoregulatory role of miR-30d-5p strengthen these observations. Overall, miRNAs may help refine patient stratification, although heterogeneity in panels studied, sampling times, and detection methods currently limits the possibility of robust synthesis and standardized application. Harmonized longitudinal studies will be necessary to confirm the predictive and mechanistic value of these signatures.

Proteomics is a promising tool for identifying predictive biomarkers of PR and monitoring the progression of T1D. DIATAG showed that considering PR as a continuous phenomenon is key to detecting protein variations that reflect the dynamics of residual β-cell function. Mass spectrometry revealed proteins and peptides associated with IDAA1c or C-peptide, such as SKAP2, CRKL, YWHAZ and various tubulins. These proteins are linked to processes governing β-cell sensitivity to inflammatory stress, immune regulation and systemic metabolic responses. The INNODIA group also used mass spectrometry to analyze the serum of newly diagnosed T1D patients to identify markers of biological changes in T1D (174, 175). Several target proteins exhibited differential expression between T1D patients and their healthy siblings. The DIATAG and INNODIA approaches, although based on distinct populations and indicators, have highlighted protein signatures associated with β-cell function decline, reinforcing the value of proteomics in characterizing the biological trajectories of T1D.

## 10 Conclusion

Our systematic review highlights the clinical factors and biomarkers associated with PR in pediatric T1D. Standard-of-care biomarkers, particularly CGM-derived measures, appear sufficient to identify PR and monitor its impact on glycemic outcomes. Biomarkers beyond routine practice allow us to explore hypothesis concerning pathophysiological mechanisms of PR and T1D, although the observational design of the studies included in this review limits causal inference. We compile all the clinical factors likely to influence the occurrence and duration of PR. We believe that their careful integration as confounding factors in clinical studies would optimize evaluations of immunotherapeutic treatments aimed at slowing the decline in β-cell function.

## Data Availability

The original contributions presented in the study are included in the article/**Supplementary Material**, further inquiries can be directed to the corresponding author/s.
