# Supplementary material for: Clinical parameters and emerging biomarkers of partial remission in pediatric type 1 diabetes: a systematic review
Source: Front Endocrinol (Lausanne). 2026 Feb 9;17:1758848. doi: 10.3389/fendo.2026.1758848 (PMC12926117; doi:10.3389/fendo.2026.1758848)
Supplement: Supplementary file 1 [file DataSheet1.docx]

Clinical parameters and emerging biomarkers of partial remission in pediatric type 1 diabetes: a systematic review

Camille Dikranian, Oumayma Hadara and Philippe A. Lysy

Supplementary Material

# Supplementary Data 1 Search strategies for each database, conducted in October 2025

**Scopus**

TITLE-ABS-KEY(("type 1 diabetes mellitus" OR "type 1 diabetes" OR "diabetes mellitus type 1" OR T1D)

AND (pediatric* OR paediatric* OR child* OR children OR adolescen* OR youth OR teen*)

AND ("new-onset" OR "new onset" OR "recent-onset" OR "recently diagnosed" OR "newly diagnosed" OR "early stage*"))

AND TITLE-ABS-KEY(("partial remission" OR "clinical remission" OR remission OR "honeymoon phase" OR IDAA1c OR IDAA1C))

AND TITLE-ABS-KEY((biomarkers OR "biological marker*" OR "predictive marker*" OR "predictive factor*" OR "immune marker*" OR "inflammatory marker*"

OR cytokine* OR chemokine* OR interleukin* OR "TNF alpha" OR "IL-6" OR "IL-10" OR "IL-17" OR "TGF-beta"

OR "regulatory T cell*" OR Treg* OR Th17 OR "immune cell*" OR lymphocyte* OR leukocyte*

OR "autoantibod*" OR autoimmun*

OR "glycemic variability" OR "glycaemic variability" OR "glycemic parameter*" OR CGM OR "continuous glucose monitoring" OR "C-peptide"

OR proteomic* OR "mass spectrometr*" OR microRNA* OR miRNA* OR hormone*))

AND (PUBYEAR > 2008 AND PUBYEAR < 2026)

AND DOCTYPE(ar)

**Embase**

('type 1 diabetes mellitus'/exp OR 'type 1 diabetes mellitus':ti,ab,kw OR 'pediatric type 1 diabetes':ti,ab,kw OR 'pediatric new onset type 1 diabetes':ti,ab,kw OR 'new-onset type 1 diabetes':ti,ab,kw OR 'children with type 1 diabetes':ti,ab,kw) AND ('partial remission':ti,ab,kw OR 'clinical remission':ti,ab,kw OR 'remission phase':ti,ab,kw OR 'partial remission phase':ti,ab,kw OR 'honeymoon phase':ti,ab,kw OR idaa1c:ti,ab,kw) AND ('biological marker'/exp OR biomarker*:ti,ab,kw OR 'predictive value'/exp OR 'predictive factor*':ti,ab,kw OR 'immune marker*':ti,ab,kw OR 'inflammatory marker*':ti,ab,kw OR 'leukocyte'/exp OR leukocyte*:ti,ab,kw OR 'lymphocyte subset'/exp OR lymphocyte*:ti,ab,kw OR 't lymphocyte regulatory' OR 'regulatory t cell*':ti,ab,kw OR treg*:ti,ab,kw OR th17:ti,ab,kw OR cytokine* OR cytokine*:ti,ab,kw OR interleukin*:ti,ab,kw OR 'tnf alpha':ti,ab,kw OR 'tgf beta':ti,ab,kw OR chemokine* OR chemokine*:ti,ab,kw OR 'autoantibody'/exp OR autoantibod*:ti,ab,kw OR autoimmun*:ti,ab,kw OR 'glycemic variability':ti,ab,kw OR 'glycemic parameter*':ti,ab,kw OR cgm:ti,ab,kw OR 'c-peptide':ti,ab,kw OR 'proteomics'/exp OR proteomic*:ti,ab,kw OR 'mass spectrometry'/exp OR 'mass spectrometr*':ti,ab,kw OR 'hormone'/exp OR hormone*:ti,ab,kw OR 'microrna'/exp OR microrna*:ti,ab,kw OR mirna*:ti,ab,kw OR 'non-coding rna':ti,ab,kw OR 'noncoding rna':ti,ab,kw) AND [humans]/lim AND [article]/lim AND [2009-2025]/py

**Pubmed**

( (pediatric*[Title/Abstract] OR paediatric*[Title/Abstract] OR child*[Title/Abstract] OR adolescent*[Title/Abstract]) AND ("type 1 diabetes"[Title/Abstract] OR "type 1 diabetes mellitus"[Title/Abstract] OR "Diabetes Mellitus, Type 1"[Mesh]) ) AND ( (partial remission[Title/Abstract]) OR (remission phase[Title/Abstract]) OR (honeymoon phase[Title/Abstract]) OR (partial remission phase[Title/Abstract]) ) AND ( (predictive markers[Title/Abstract]) OR (biomarkers[Title/Abstract]) OR (Biological Factors[MeSH Terms]) OR (factors[Title/Abstract]) OR ("Leukocytes"[Mesh]) OR ("T-Lymphocytes"[Mesh]) OR (glycemic variability[Title/Abstract]) OR (glycemic parameters[Title/Abstract]) OR (proteomics[Title/Abstract]) OR (autoantibodies[Title/Abstract]) OR (autoimmunity[Title/Abstract]) OR ("Cytokines"[MeSH Terms]) OR ("Chemokines"[MeSH Terms]) OR ("Hormones"[MeSH Terms]) OR (microRNA*[Title/Abstract]) OR (miRNA*[Title/Abstract]) OR ("MicroRNAs"[Mesh]) )

# Supplementary Table 1 Studies included in the systematic review

BMD: bone mineral density; BMI: body mass index; CGM: continuous glucose monitoring; CLIA: chemiluminescence immunoassay; cOC: carboxylated osteocalcin; DCCT: diabetes control and complications trial; DKA: diabetic ketoacidosis; DPD: deoxypyridinoline; DXA: dual energy X-ray absorptiometry; ELISA: enzyme-linked immunosorbent assay; F: female; GLP-1: glucagon-like peptide-1; HbA1c: hemoglobin A1c; HDL: high-density lipoprotein; HLA: human leukocyte antigen; HCLC : high capacity liquid chromatography; HPLC: high performance liquid chromatography; IDAA1c: insulin-dose adjusted A1C; IQR: interquartile range; LC-MS/MS: liquid chromatography- tandem mass spectrometry; LDL: low-density lipoprotein; M: male; MMTT: mixed-meal tolerance test; miRNA: micro ribonucleic acid; NGS: next-generation sequencing; OCN: osteocalcin; OPG: osteoprotegerin; P1NP: procollagen type 1 N propeptide; PR: partial remission; RANKL: receptor activator of nuclear factor- κB ligand; RIA: radioimmunoassay; RT-qPCR: reverse transcription quantitative real-time polymerase chain reaction; SD: standard deviation; TBLH: total body less head;T1D: type 1 diabetes; TG: triglyceride; TGF: transforming growth factor; ucOC: undercarboxylated osteocalcin.

| Reference | Study design | Study population (n, follow-up, age) | Definition of partial remission | Biomarker identification method |
| --- | --- | --- | --- | --- |
| *DEFINITIONS OF PR* |  |  |  |  |
| *New definition for partial remission period in children/adolescents with type 1 diabetes,* Mortensen HB et al., 2009 | Prospective multicenter cohort | n=275  Mean age at diagnosis: 9.1±3.7 years  Follow up at 1, 3, 6, 9, and 12 months after diagnosis | IDAA1c ≤9 | Fluoroimmunometric assay: stimulated C-peptide  Automatic high-pressure liquid chromatography: HbA1c |
| *Partial remission definition: validation based on the insulin dose-adjusted HbA1c (IDAA1c) in 129 Danish children with new-onset type 1 diabetes*, Andersen et al., 2014 | Prospective multicenter cohort | n=129  Estimated mean age at diagnosis : 9.7 years  Follow-up at 1, 3, 6, 9, and 12 months | IDAA1c ≤9 | Routine HbA1c measurement (HPLC) + reported daily insulin requirement → composite score IDAA1c  Fluoroimmunometric assay: stimulated C-peptide |
| *CGM DATA* |  |  |  |  |
| *Clinically serious hypoglycemia is rare and not associated with time-in-range in youth with new-onset type 1 diabetes*, Addala et al., 2021 | Prospective observational cohort | n=80  Mean age at diagnosis: 8.8±4.6 years  Follow-up at 1, 3, 6, 9, and 12 months after diagnosis | IDAA1c <9 | CGM analysis (time-in-range, severe hypoglycemia according to DCCT criteria) |
| *Glycemic Variability Patterns Strongly Correlate With Partial Remission Status in Children With Newly Diagnosed Type 1 Diabetes*, Pollé OG et al., 2022 | Prospective longitudinal cohort | n=78  Mean age at diagnosis: 10.4±3.6 years  Follow-up every 3 months for 12 months | IDAA1c <9 | CGM  Estimates of residual β-cell secretion (CLIA after glucagon stimulation test)  Clinical parameters |
| *Integration of Routine Parameters of Glycemic Variability in a Simple Screening Method for Partial Remission in Children with Type 1 Diabetes*, Nielens N et al., 2018 | Retrospective longitudinal cohort | n=239  Mean age at diagnosis: 9.1±3.8 years  Observation length: 36 months after diagnosis (visits every 3 months) | IDAA1c ≤9 | Parameters of glycemic variability  Clinical parameters  AutoDELFIA immunoassay: postprandial C-peptide  HCLC: HbA1c |
| *Post-Hypoglycemic hyperglycemia are highly relevant markers for stratification of glycemic variability and partial remission status of pediatric patients with new-onset type 1 diabetes*, Harvengt AA et al., 2023 | Prospective longitudinal cohort (including data from the multicenter DIATAG study) | n=194  Mean age at diagnosis: 10.7±3.4 years  Follow up every 3 months for 12 months | IDAA1c <9 | CGM and PHH detection  Clinical parameters  Chemiluminescence immunoassay: Residual β-cell secretion  Unsupervised hierarchical clustering: glucotypes |
| *ANTHROPOMETRIC DATA/CLINICAL FACTORS* |  |  |  |  |
| *A predictive model for lack of partial clinical remission in new-onset pediatric type 1 diabetes*, Marino KR et al., 2017 | Prospective longitudinal cohort | n=204  Mean age at diagnosis for remitters (n=86): 9.1±3.0 years Mean age at diagnosis for non-remitters (n=118): 7.0±3.1 years  Follow up every 3 months for the first year, and every 3 to 6 months up to 36 months | IDAA1c ≤9 | Clinical parameters: number of positive auto-antibodies, age, bicarbonate at diagnosis, sex, BMI |
| *Body mass index and partial remission in 119 children with type 1 diabetes — a 6-year observational study,* Sokołowska-Gadoux M. et al., 2023 | Retrospective longitudinal cohort | n=119  Mean age at diagnosis : 7.0±8.9 years  Observation length: 72 months | Insulin requirements <0.5 IU/kg BW/day and HbA1c <7% | Immunoassay: C-peptide  Anthropometric: BMI z-score |
| *Characteristics and Determinants of Partial Remission in Children with Type 1 Diabetes Using the Insulin-Dose-Adjusted A1C Definition*, Pecheur A. et al, 2014 | Retrospective observational cohort | n=242 children with new-onset T1D  Mean age at diagnosis: 8.8±3.8 years (range 0.9–16.4)  Observation length: up to 60 months (regular visits every 3 months) | IDAA1c ≤9 | AutoDELFIA immunoassay: postprandial C-peptide  HPLC for HbA1c  Clinical data: insulin and DKA |
| *Early weight gain after diagnosis may have an impact on remission status in children with new-onset type 1 diabetes mellitus*, Emet et al., 2023 | Retrospective observational cohort | n=99  Mean age at diagnosis: 8.7±3.6 years  Observation length: 12 months after diagnosis | IDAA1c ≤9 | Clinical and anthropometric parameters: BMI-SDS variation  HbA1c: HPLC |
| *Factors contributing to partial remission in type 1 diabetes: analysis based on the insulin dose-adjusted HbA1c in 3657 children and adolescents from Germany and Austria*, Nagl K et al., 2017 | Retrospective multicenter cohort | n=3,657  Estimated mean age at diagnosis: 7.7 years  Observation length: 72 months after diagnosis (evaluated timepoints: 1, 3, 6, 12, 18, 24, 36, 48, and 72 months) | IDAA1c ≤9 | Clinical parameters  HbA1c (standardized to DCCT reference via multiple-of-the-mean method) and daily insulin dose  C-peptide: immunoassay  Autoantibodies (ICA, IAA, GADA, IA-2A, ZnT8A): immunoassays |
| *Features of partial remission in children with* *type 1 diabetes using the insulin dose-adjusted A1c definition and risk factors associated with,* Wong T.W.C. et al., 2021 | Retrospective observational cohort | n=57  Mean age at diagnosis: 10.1±3.9 years  Observation length: 24 months | IDAA1c ≤9 | HbA1c and daily insulin dose used to calculate IDAA1c |
| *Frequency, clinical characteristics, and determinants of partial remission in type 1 diabetes: Different patterns in children and adults*, Zhong T. et al., 2020 | Prospective observational cohort | n=300 (186 children, 114 adults)  Mean age at diagnosis: 9.9±4.3 years for children; 33.6±12.0 years for adults  Follow-up: median 24 months (range 12–56 months) | Stimulated C-peptide ≥300 pmol/L  or  IDAA1c ≤9 | Chemiluminescence immunoassay: C-peptide via MMTT  Radioimmunoassay: GADA, IA-2A, ZnT8A autoantibodies |
| *Influence of age on partial clinical remission among children with newly diagnosed type 1 diabetes*, Passanisi et al., 2020 | Prospective observational cohort | n=167  Mean age at diagnosis: 13.8±4.1 years  Follow-up at 3, 6, 9, 12, 15, 18, and 24 months post diagnosis | IDAA1c ≤9 | HPLC: HbA1c Daily insulin Immunoassay: C-peptide |
| *Markers influencing the presence of partial clinical remission in patients with newly diagnosed type 1 diabetes*, Pyziak A. et al., 2017 | Retrospective observational cohort | n=186 children  Estimated mean age at diagnosis: 9.41 years  Observation length: 24 months after diagnosis (evaluated time points: 3, 6, 12, 18, and 24 months) | Insulin requirements <0.5 IU/kg BW/day and HbA1c <7% | Electrochemiluminescence immunoassay: Fasting C-peptide  HPLC: HbA1c  Lipid profile: total cholesterol, HDL, LDL, and TG by enzymatic colorimetric assay |
| *Partial remission in Brazilian children and adolescents with type 1 diabetes. Association with a haplotype of class II human leukocyte antigen and synthesis of autoantibodies*, Camilo DS et al., 2020 | Prospective longitudinal cohort | n=51 with new-onset T1D  Estimated mean age at diagnosis = 11.8 years  Median time follow-up of 13 months from diagnosis | IDAA1c ≤9 | HLA genotyping  Clinical parameters  HCLC: HbA1c  CLIA: C-peptide |
| *Partial remission in type 1 diabetes and associated factors: Analysis based on the insulin dose-adjusted hemoglobin A1c in children and adolescents from a regional diabetes center, Auckland, New Zealand*, Chiavaroli et al., 2019 | Retrospective observational cohort | n=614  Mean age at diagnosis: 8.5 years  Observation length : 18 months (evaluated timepoints: 3, 6, 12 and 18 months) | IDAA1c ≤9 | HbA1c (HPLC) + daily insulin requirement → IDAA1c calculation |
| *Racial/ethnic minority youth with recent-onset type 1 diabetes have poor prognostic factors*, Redondo et al., 2018 | Prospective longitudinal cohort | n=927  Mean age at diagnosis: 9.23±4.2 years  Median follow-up: 36 months | IDAA1c ≤9 | Immunoassay: HbA1c Immunoenzymometric assay (Tosoh): daily insulin, C-peptide |
| *Regular physical activity as a physiological factor contributing to extend partial remission time in children with new onset diabetes mellitus*, Jamiołkowska-Sztabkowska et al., 2020 | Prospective longitudinal cohort | n=125  Median age: 10 years  Estimated mean age at diagnosis : 9.8 years  Follow-up at 3, 6, 12, 24 months after diagnosis | Insulin requirements <0.5 IU/kg BW/day and HbA1c <7% | HPLC: HbA1c Immunoassay: C-peptide Validated physical activity questionnaire |
| *Remission phase in children diagnosed with type 1 diabetes in years 2012 to 2013 in Silesia, Poland: An observational study*, Chobot et al., 2019 | Retrospective multicenter observational cohort | n=194  Mean age at diagnosis : 8.1±4.3 years  Observation length: 48 months after diagnosis | IDAA1c ≤9 | Routine HbA1c measurement + insulin dose → IDAA1c calculation |
| *HORMONES* |  |  |  |  |
| *Bone turnover markers during the remission phase in children and adolescents with type 1 diabetes*, Madsen JOB et al., 2020. | Prospective cohort | n=99  Mean age at diagnosis =11.0±3.6  Follow-up at 6 and 12 months after diagnosis | IDAA1c ≤9  Or  stimulated C-peptide >300 pmol/L | OCN, P1NP → bone formation markers  CTX → bone resorption marker  Methods: automated immunoassays iSYS (IDS-iSYS, Immunodiagnostic Systems), CLIA |
| *Metabolic bone markers can be related to preserved insulin secretion in children with newly diagnosed type 1 diabetes*, Szymańska M. et al., 2020 | Prospective longitudinal cohort | n=100 children with new-onset T1D  n=52 healthy subjects  Estimated mean age = 11.4±3.2 years  Mean follow-up 7 months (6–8 months). | Insulin requirements <0.5 IU/kg BW/day and HbA1c <7% | Electrochemiluminescence immunoassay: fasting and glucagon-stimulated C-peptide  ELISA: OPG and s-RANKL  Chemiluminescence: urinary DPD  HPLC: HbA1c  DXA: BMD (TBLH, L1–L4). |
| Proinsulin, GLP-1, and glucagon are associated with partial remission in children and adolescents with newly diagnosed type 1 diabetes, Kaas A et al., 2012 | Prospective cohort  (The Hvidoere Remission Phase Study cohort) | n=275  Mean age at diagnosis: 9.1 (0.2-16.8) years  Group of patients <5 years old (n=48) = mean age at diagnosis: 3.1 (0.21-4.9) years  Group of patients between 5 and 10 years of age = mean age 7.8 (5.1-9.9) years  Group of patients ≥10 years old = mean age: 12.4 (10.0-16.8)  Follow-up 1, 6, and 12 months after diagnosis | IDAA1c ≤9 | MMTT: proinsulin, C-peptide Fluoroimmunoassay: serum C-peptide ELISA: proinsulin RIA: GLP-1, glucagon HPLC: HbA1c |
| *Serum C-peptide and osteocalcin levels in children with recently diagnosed diabetes*, Sabek et al., 2020 | Prospective longitudinal cohort | n=68 (including 48 with T1D)  Estimated mean age: 11.9 (estimated SD 3.0) years  Follow-up: 40.4 months | IDAA1c ≤9 | Immunoassay: Serum C-peptide, proinsulin, cOC and uOC |
| *VARIATIONS IN IMMUNE CELL SUBSETS/CYTOKINES* |  |  |  |  |
| *A pilot study showing associations between frequency of CD4^+^ memory cell subsets at diagnosis and duration of partial remission in type 1 diabetes,* Moya R. et al., 2016 | Retrospective observational cohort | n=19  Age between 9 and 16 years.  Observation length: 24 months after diagnosis (evaluated timepoints: 3, 6, 9, 12, 18, 24 months) | IDAA1c ≤9 | Flow cytometry: CD4 memory subsets  Immunoassay: C-peptide, HbA1c |
| *Candidate Biomarkers for the Prediction and Monitoring of Partial Remission in Pediatric Type 1 Diabetes*, Gomez-Muñoz L et al., 2022 | Prospective longitudinal cohort | n=17 with new-onset T1D and 17 sex-matched controls + 10 pediatric patients at T1D onset for PR predictive model  Mean age at T1D onset: 8.7±3.6 years  Follow-up: blood samples at disease onset, at PR or 8 months for non-remitters and 12 months after disease onset | IDAA1c ≤9 | Flow cytometry Clinical parameters HPLC: HbA1c  ELISA: fasting basal C-peptide Glucagon test: stimulated C-peptide (measured 6 min after administration)  Serum cytokine quantification |
| *Changes in innate and adaptive immunity over the first year after onset of T1D,* Klocperk A. et al, 2020 | Prospective observational cohort | n=38  Mean age: 9.4±4.0 years.  Follow-up: 12 months (baseline, 6, and 12 months) | IDAA1c ≤9 | Electrochemiluminescence immunoassay: C-peptide by MMTT  Flow cytometry: T, B, dendritic cell subsets |
| *Combined unsupervised and semi-automated supervised analysis of flow cytometry data reveals cellular fingerprint associated with newly diagnosed pediatric type 1 diabetes*, Bechi Genzano C et al., 2022 | Prospective observational cohort | n=107 with new onset T1D, 85 relatives with 0-1 islet autoantibodies, 58 patients with celiac disease or autoimmune thyroiditis, and 76 healthy controls  Estimated mean age at onset for T1D patients: 11.1 years  Median follow up: 12.2 (IQR 11.1-13.2) months | IDAA1c ≤9 | Clinical parameters Flow cytometry Unsupervised clustering and semi-automated supervised gating |
| *Immune cell and cytokine patterns in children with type 1 diabetes mellitus undergoing a remission phase: A longitudinal study*, Fitas AL et al., 2018 | Prospective longitudinal cohort | n=28  Mean age at T1D onset: 10.0±2.6 years  Follow up: T1 (disease onset), T2 (PR), T3 (IDAA1c >9) | IDAA1c ≤9 | Flow cytometry: characterization of immune cell subsets  Cell stimulation: intracellular cytokine evaluation Luminex technology: serum cytokine quantification  Chromatography: HbA1c  Chemiluminescence: C-peptide and 25OHD |
| *Immunological Balance Between Treg and Th17 Lymphocytes as a Key Element of Type 1 Diabetes Progression in Children*, Starosz A. et al., 2022 | Prospective longitudinal study | n=60 children with newly diagnosed T1D; control group n=31  Estimated mean age at onset : 11.6±3.3 years  Follow-up: 24 months (visits at 3, 6, 12, and 24 months) | Insulin requirements <0.5 IU/kg BW/day and HbA1c <7% | Flow cytometry for Treg: CD4⁺CD25⁺CD127⁻FoxP3⁺ and Th17 (IL-17A⁺) cells  ELISA for IL-10 and IL-17 cytokines  RT-qPCR for gene expression: CTLA4, CD28, PPARG, RRAD  Immunoassay for fasting C-peptide: ECLIA |
| *NK Cell Subsets Changes in Partial Remission and Early Stages of Pediatric Type 1 Diabetes*, Gomez-Muñoz L et al., 2021 | Prospective longitudinal cohort | n=17 with new-onset T1D and 17 sex-matched controls Mean age at T1D onset: 8.7±3.6 years Follow-up: blood samples at disease onset, at PR or 8 months for non-remitters and 12 months after disease onset | IDAA1c ≤9 | Flow cytometry  Clinical parameters  HPLC: HbA1c  ELISA: fasting basal C-peptide HLA class II typing |
| *Partial remission and early stages of pediatric type 1 diabetes display immunoregulatory changes. A pilot study*, Villalba A et al., 2019 | Prospective longitudinal cohort | n=52 with T1D (30 age-matched controls) Mean age at T1D onset (n=22): 9.1±4.5 years Follow up: T1D onset, +6, +12, +18 months | Insulin requirements <0.5 IU/kg BW/day and HbA1c <7%  OR IDAA1c <9 | ELISA: plasma concentrations of TGF, betatrophin, HLA-G Flow cytometry: analysis of cellular subsets Quantitative RT-PCR |
| *Systemic TNFα correlates with residual β-cell function in children and adolescents newly diagnosed with type 1 diabetes*, Overgaard A.J. et al., 2020 | Prospective longitudinal study | n=63 children/adolescents (33% F)  Mean age: 11.3±3.7 years (range 3.3–17.7)  Follow-up: 12 months (sampling at baseline, 6, and 12 months). | Insulin requirements <0.5 IU/kg BW/day and HbA1c <7% | ECLIA: fasting and 6-min glucagon-stimulated C-peptide and osteocalcin  ELISA: OPG and s-RANKL  Chemiluminescence: urinary DPD  DXA: BMD  HPLC: HbA1c |
| *Fetuin A and Interleukin 8 in Children with Clinical Remission of Type 1 Diabetes*, Pyziak-Skupien A. et al., 2020 | Prospective longitudinal cohort | n=134 children with new-onset T1D  Control group n=47 healthy children  Mean age: 9.9±3.1 years  Follow-up: 24 months (at 3, 6, 12, 18, and 24 months) | IDAA1c <9 | ELISA immunoassay: fetuin-A and IL-8  HPLC: HbA1c  Immunofluorescence and ELISA: Islet auto-antibodies GADA, ZnT8, ICA |
| *HLA GENOTYPING* |  |  |  |  |
| *The Unfavorable Impact of DR9/DR9 Genotype on Partial Remission in T1D,* Chen Y. et al, 2022 | Prospective longitudinal observational cohort | n=237 (68.8% childhood-onset)  Mean age at diagnosis: 12.6±5.4 years.  Follow-up: 24 months | Stimulated C-peptide ≥300 pmol/L | Chemiluminescence immunoassay: C-peptide by MMTT  Radioligand assays: GADA, IA-2A, ZnT8A.  HLA-DR/DQ genotyping |
| *Partial remission in Brazilian children and adolescents with type 1 diabetes. Association with a haplotype of class II human leukocyte antigen and synthesis of autoantibodies*, Camilo DS et al., 2020 | Prospective longitudinal cohort | n=51 with new-onset T1D  Estimated mean age at diagnosis = 11.8 years  Median time follow-up of 13 months from diagnosis | IDAA1c ≤9 | HLA genotyping  Clinical parameters  HCLC: HbA1c  CLIA: C-peptide |
| *MiRNAs* |  |  |  |  |
| *Circulating microRNA levels predict residual beta cell function and glycaemic control in children with type 1 diabetes mellitus,* Samandari et al., 2017 | **Discovery study** Prospective longitudinal cohort  **Validation study** Prospective observational cohort (validation of candidate miRNAs in remaining cohort, measured 3 months after diagnosis) | The Danish Remission Phase Cohort  **Discovery cohort** n=40  Mean age at diagnosis: 8.7±3.4 years Follow up at 1, 3, 6, 12, and 60 months post diagnosis  **Validation cohort** n=80 Mean age at diagnosis: 10.7±4.1 years Follow up at 1, 3, 6, 12, and 60 months post diagnosis | IDAA1c ≤9 | miRNA isolation and reverse transcription-quantitative PCR   Statistical analysis and identification of candidate miRNAs  Clinical parameters |
| *Immunoregulatory biomarkers of the remission phase in Type 1 diabetes: miR-30d-5p modulates PD-1 expression and regulatory T cell expansion*, Gomez-Muñoz et al., 2023 | Prospective observational cohort | **Discovery cohort** n=17 newly diagnosed with T1D (mean age: 8.7±3.6 years), including 11 remitters (9.1±4.3 years), and six non-remitters (at 8 months after diagnosis; 9±2.8 years), plus 17 age- and sex-matched controls (8.8±3.4 years)  Follow-up at disease onset and at PR or 8 months for non-remitters  **Validation cohort** 15 age- and sex-matched non-diabetic controls (9.7±3.6 years), eight newly diagnosed with T1D (11.6±2.7 years), 10 remitters (11.8±2.9 years), and 9 non-remitters (7.3±3.7 years) | IDAA1c ≤9 | Silica column adsorption and elution: isolation of circulating RNAs (including microRNAs) from serum/plasma  NGS Determination of gene targets for miRNAs Determination of gene ontology and pathway analysis Quantitative RT-qPCR Flow cytometry: percentage of immune cell subpopulations |
| *Influence of disease duration on circulating levels of miRNAs in children and adolescents with new onset type 1 diabetes*, Samandari et al., 2018 | Prospective longitudinal cohort | n=40  Mean age at diagnosis: 8.7 (SD 3.4) years  Follow-up at 1, 3, 6, 12, 60 months after diagnosis | IDAA1c ≤9 | qRT-PCR: circulating plasma microRNAs |
| *PROTEOMICS* |  |  |  |  |
| *Plasma proteomics in children with new-onset type 1 diabetes identifies new potential biomarkers of partial remission*, Pollé OG et al., 2024 | Prospective observational cohort | n=16  Mean age at T1D onset: 9.1±4.2 years Remitters (n=8): 11.7±2.8 years Non remitters (n=8): 6.5±3.8 years  Follow-up: from disease onset to + 3 months | IDAA1c <9 | LC-MS/MS: proteomics (relative quantification)  Clinical parameters Estimated β-cell secretion |

**Supplementary Table 2** Joanna Briggs Institute Critical Appraisal Checklist

| Study identification | 1. Similar groups/same population? | 2. Exposures measured similarly? | 3. Exposure valid/reliable? | 4. Confounders identified? | 5. Confounding strategies? | 6. Outcome absent at baseline? | 7. Outcomes valid/reliable? | 8. Follow-up sufficient? | 9. Follow-up complete/reasons? | 10. Strategies for incomplete follow-up? | 11. Appropriate statistical analysis? |
| --- | --- | --- | --- | --- | --- | --- | --- | --- | --- | --- | --- |
| Camilo et al., 2020 | N/A | Yes | Yes | Unclear | No | Yes | Yes | Yes | Unclear | No | Yes |
| Gomez-Muñoz et al., 2022 | Yes | Yes | Yes | Unclear | Yes | Yes | Yes | Yes | Unclear | No | Yes |
| Samandari et al., 2018 | Yes | Yes | Yes | Yes | Yes | Yes | Yes | Yes | Unclear | Unclear | Yes |
| Villalba et al., 2019 | Yes | Yes | Yes | Unclear | No | Yes | Yes | Yes | Unclear | No | Yes |
| Bechi Genzano et al., 2022 | Yes | Yes | Yes | Yes | Yes | Yes | Yes | Yes | Unclear | Unclear | Yes |
| Pollé et al., 2024 | Yes | Yes | Yes | Unclear | Yes | Yes | Yes | Yes | Unclear | No | Yes |
| Pollé et al., 2022 | Yes | Yes | Yes | Yes | Yes | Yes | Yes | Yes | Unclear | Unclear | Yes |
| Harvengt et al., 2023 | Yes | Yes | Yes | Yes | Yes | Yes | Yes | Yes | Unclear | Unclear | Yes |
| Gomez-Muñoz et al., 2021 | Yes | Yes | Yes | Yes | Yes | Yes | Yes | Yes | No | Unclear | Yes |
| Chiavaroli et al., 2019 | Yes | Yes | Yes | Yes | Yes | Yes | Yes | Yes | Unclear | Unclear | Yes |
| Madsen et al., 2020 | Yes | Yes | Yes | Yes | Yes | Yes | Yes | Yes | Unclear | Unclear | Yes |
| Nagl et al., 2017 | Yes | Yes | Yes | Yes | Yes | Yes | Yes | Yes | Unclear | No | Yes |
| Nielens et al., 2018 | Yes | Yes | Yes | Yes | Yes | Yes | Yes | Yes | No | No | Yes |
| Andersen et al., 2014 | Yes | Yes | Yes | Yes | Yes | Yes | Yes | Yes | Unclear | No | Yes |
| Samandari et al., 2017 | Yes | Yes | Yes | Yes | Yes | Yes | Yes | Yes | Yes | Unclear | Yes |
| Fitas et al., 2018 | Yes | Yes | Yes | Unclear | No | Yes | Yes | Yes | Yes | No | Unclear |
| Kaas et al., 2012 | Yes | Yes | Yes | Yes | Yes | Yes | Yes | Yes | Yes | Unclear | Yes |
| Addala et al., 2021 | Yes | Yes | Yes | Yes | Yes | Yes | Yes | Yes | Yes | Unclear | Yes |
| Gomez-Muñoz et al., 2023 | Unclear | Yes | Yes | Unclear | Unclear | Yes | Yes | Yes | Yes | Unclear | Yes |
| Passanisi et al., 2020 | Yes | Yes | Yes | Yes | Yes | Yes | Yes | Yes | Yes | Unclear | Yes |
| Marino et al., 2017 | Yes | Yes | Yes | Yes | Yes | Yes | Yes | Yes | Yes | Unclear | Yes |
| Redondo et al., 2018 | Unclear | Yes | Yes | Yes | Yes | Yes | Yes | Yes | Unclear | Unclear | Yes |
| Jamiołkowska-Sztabkowska et al., 2020 | Yes | Yes | Unclear | Yes | Unclear | Yes | Yes | Yes | Yes | Unclear | Yes |
| Sabek et al., 2020 | Unclear | Yes | Yes | Yes | Unclear | Yes | Yes | Yes | Unclear | Unclear | Yes |
| Chobot et al., 2019 | Yes | Yes | Yes | Yes | Unclear | Yes | Yes | Yes | Unclear | Unclear | Yes |
| Emet et al., 2023 | Yes | Yes | Yes | Yes | Unclear | Yes | Yes | Yes | Unclear | Unclear | Yes |
| Mortensen et al., 2009 | Yes | Yes | Yes | Yes | Unclear | Yes | Yes | Yes | Unclear | Unclear | Yes |
| Sokolowska-Gadoux et al., 2023 | Yes | Yes | Yes | Yes | Unclear | Yes | Yes | Yes | Yes | Unclear | Yes |
| Pecheur et al., 2014 | Yes | Yes | Yes | Yes | Yes | Yes | Yes | Yes | Unclear | Unclear | Yes |
| Wong et al., 2021 | Yes | Yes | Yes | Yes | Yes | Yes | Yes | Yes | Yes | Unclear | Yes |
| Zhong et al., 2020 | Yes | Yes | Yes | Yes | Yes | Yes | Yes | Yes | Unclear | Unclear | Yes |
| Pyziak et al., 2017 | Yes | Yes | Yes | Yes | Yes | Yes | Yes | Yes | Unclear | Unclear | Yes |
| Pyziak-Skupien et al., 2020 | Yes | Yes | Yes | Yes | Yes | Yes | Yes | Yes | Unclear | Unclear | Yes |
| Szymanska et al., 2020 | Yes | Yes | Yes | Yes | No | Yes | Yes | Unclear | Unclear | Unclear | Unclear |
| Overgaard et al., 2020 | Yes | Yes | Yes | Yes | Yes | Yes | Yes | Yes | Yes | Unclear | Yes |
| Moya et al., 2016 | Unclear | Yes | Yes | Yes | Yes | Yes | Yes | Yes | Unclear | Unclear | Yes |
| Klocperk et al., 2020 | Yes | Yes | Yes | Yes | Yes | Yes | Yes | Yes | Unclear | Unclear | Yes |
| Starosz et al., 2022 | Unclear | Yes | Yes | Yes | No | Yes | Yes | Yes | Unclear | No | Yes |
| Chen et al., 2022 | Yes | Yes | Yes | Unclear | No | Yes | Yes | Yes | Unclear | Unclear | Yes |
